# Dynamic Methylation of an L1 Transduction Family during Reprogramming and Neurodifferentiation

**DOI:** 10.1128/MCB.00499-18

**Published:** 2019-03-19

**Authors:** Carmen Salvador-Palomeque, Francisco J. Sanchez-Luque, Patrick R. J. Fortuna, Adam D. Ewing, Ernst J. Wolvetang, Sandra R. Richardson, Geoffrey J. Faulkner

**Affiliations:** a Mater Research Institute, University of Queensland, Woolloongabba, Queensland, Australia; b Pfizer-University of Granada-Andalusian Government Center for Genomics and Oncological Research, Granada, Spain; c Australian Institute for Bioengineering and Nanotechnology, University of Queensland, St. Lucia, Queensland, Australia; d Queensland Brain Institute, University of Queensland, Brisbane, Queensland, Australia; e School of Biomedical Sciences, University of Queensland, Brisbane, Queensland, Australia

**Keywords:** L1, LINE-1, methylation, neurogenesis, retrotransposon

## Abstract

The retrotransposon LINE-1 (L1) is a significant source of endogenous mutagenesis in humans. In each individual genome, a few retrotransposition-competent L1s (RC-L1s) can generate new heritable L1 insertions in the early embryo, primordial germ line, and germ cells. L1 retrotransposition can also occur in the neuronal lineage and cause somatic mosaicism. Although DNA methylation mediates L1 promoter repression, the temporal pattern of methylation applied to individual RC-L1s during neurogenesis is unclear. Here, we identified a *de novo* L1 insertion in a human induced pluripotent stem cell (hiPSC) line via retrotransposon capture sequencing (RC-seq). The L1 insertion was full-length and carried 5ʹ and 3ʹ transductions. The corresponding donor RC-L1 was part of a large and recently active L1 transduction family and was highly mobile in a cultured-cell L1 retrotransposition reporter assay. Notably, we observed distinct and dynamic DNA methylation profiles for the *de novo* L1 and members of its extended transduction family during neuronal differentiation. These experiments reveal how a *de novo* L1 insertion in a pluripotent stem cell is rapidly recognized and repressed, albeit incompletely, by the host genome during neurodifferentiation, while retaining potential for further retrotransposition.

## Abstract (precis)

The retrotransposon LINE-1 (L1) is a significant source of endogenous mutagenesis in humans. In each individual genome, a few retrotransposition-competent L1s (RC-L1s) can generate new heritable L1 insertions in the early embryo, primordial germ line, and germ cells.

## INTRODUCTION

LINE-1 (L1) retrotransposons are mobile genetic elements that occupy nearly 20% of the human genome (1) and are an endogenous source of mutagenesis (2). Approximately 100 retrotransposition-competent L1s (RC-L1s) are found in each individual, while the remaining ∼500,000 L1 copies are immobile due to 5′ truncations, inversions, deletions, and other mutations (3, 4). Almost all RC-L1s belong to the L1-Ta subfamily (5). L1 retrotransposition is a copy-and-paste process involving an RNA intermediate and an L1-encoded protein machinery (6–8) that orchestrates L1 integration via a molecular process termed target-primed reverse transcription (TPRT) (9). An RC-L1 is 6 kb in length and contains a 5′ untranslated region (UTR), an antisense promoter and open reading frame (ORF0) (10, 11), two nonoverlapping sense open reading frames (ORF1 and ORF2), and a 3′ UTR that is punctuated by a poly(A) tract (12–14). Critically, ORF2p possesses endonuclease (EN) and reverse transcriptase (RT) activities required for L1 mobility (8, 15, 16), while new L1 insertions usually integrate at a degenerate L1 EN recognition motif (5ʹ-TT/AAAA, where “/” represents the position cut by the L1 EN) (17) and are flanked by variable-length target site duplications (TSDs) (18, 19), which are hallmarks of TPRT. L1 is the only active autonomous human retrotransposon (5, 15) although other polyadenylated RNAs, including mRNAs and those of the Alu and SINE-VNTR-Alu (SVA) retrotransposon families, can be mobilized in *trans* by the L1 machinery (13, 20–24). The L1 5′ UTR has an internal RNA polymerase II promoter that directs L1 mRNA transcription (25) and is regulated by DNA methylation of a CpG island located nearby in the 5′ UTR (26–31). The host genome also restricts L1 activity through mechanisms limiting L1 mRNA production or otherwise hindering retrotransposition (32–34).

A minor fraction of RC-L1s in the human population are thought to generate the majority of new germ line L1 insertions and are highly mobile, or “hot,” when tested in cultured cell L1 retrotransposition assays (3, 8, 35–37). These experiments largely measure the enzymatic efficiency of L1s introduced in episomal vectors, and, importantly, a particular L1 locus may present multiple alleles with different retrotransposition efficiencies (38, 39). The endogenous regulation of a given RC-L1 may therefore be most clearly resolved in the spatiotemporal contexts where it produces new L1 insertions. An RC-L1 can be identified as the donor element for an L1 insertion through shared unique internal single nucleotide variants, or transductions (37, 40). 5ʹ transductions are thought to accompany <0.1% of L1-Ta insertions and likely arise when the L1 promoter, or another nearby promoter, initiates L1 mRNA transcription upstream of the canonical L1 transcription start site (1, 32, 41–43). In contrast, 3ʹ transductions are found alongside ∼20% of new germ line L1 insertions and occur when L1 mRNA transcription bypasses the canonical L1 polyadenylation signal and terminates at an alternative downstream polyadenylation signal (1, 13, 44–48). Transductions have been used to trace RC-L1s responsible for pathogenic L1 insertions (32, 35, 49–53) and to reconstruct closely related RC-L1 lineages, or transduction families, in human populations (35, 44, 54).

Early embryogenesis provides a major developmental niche for heritable L1 retrotransposition events in mammals (55–57). Cultivated human embryonic stem cells (hESCs) and human induced pluripotent stem cells (hiPSCs) resembling the cells of the embryonic inner cell mass also express L1 mRNAs and support engineered and endogenous L1 retrotransposition (58–63). *De novo* L1 insertions arising during embryogenesis or later development can cause somatic mosaicism (55, 64–66). In particular, somatic L1 insertions have been reported in brain tissue (65, 67–74), while engineered L1 reporter genes mobilize during neurogenesis and in postmitotic neurons (63, 67, 75). Importantly, the L1-Ta subfamily is hypomethylated in hESCs and hiPSCs compared to methylation of neurons and other differentiated cells, suggesting genome-wide L1 promoter methylation is enforced during development (58, 61, 63, 67). However, the likely related temporal profiles of DNA methylation and somatic retrotransposition for individual RC-L1s that mobilize during neurogenesis are unresolved.

Here, we identified a reprogramming-associated *de novo* L1 insertion in a cultivated hiPSC line. This insertion was traced to a hot donor RC-L1 that was part of an extended and recently active transduction family. We then measured locus-specific DNA methylation among *de novo*, donor, and transduction family L1 promoters, as well as the L1-Ta subfamily genome-wide, at multiple points of neurodifferentiation. These experiments significantly elucidate the dynamic temporal profile of epigenetic L1 repression applied to new and extant L1 insertions during neurogenesis.

## RESULTS

### A *de novo* L1 insertion arising during reprogramming.

**FIG 1:**
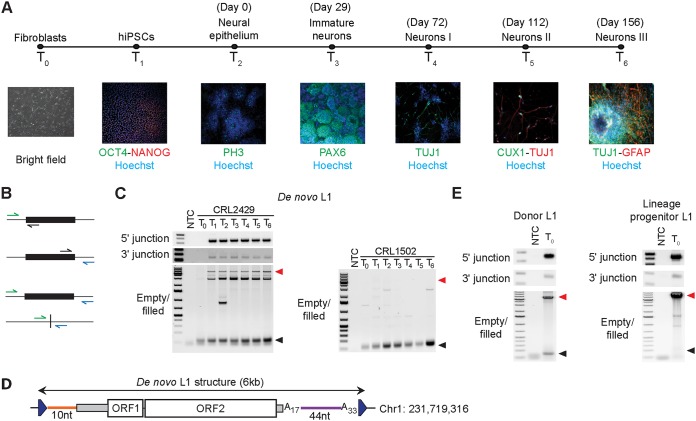
Characterization of a reprogramming-associated *de novo* L1 insertion carried through neurodifferentiation *in vitro*. (A) Schematic timeline of experimental approach. Fibroblasts (time point 0 [*T*_0_]) were reprogrammed to obtain hiPSCs (*T*_1_), which were then sampled at 5 points (*T*_2_ to *T*_6_) of neuronal differentiation in extended cell culture. Immunocytochemistry was used to characterize expression of marker genes (OCT4, NANOG, PAX6, TUJ1, CUX1, and GFAP gemes) and histone 3 phosphorylation (PH3), as associated with various stages of neural cell maturation, with Hoechst staining of DNA. (B) L1 insertion PCR validation strategies. Green and blue arrows, respectively, represent primers targeting the 5′ and 3′ genomic flanks of an L1 insertion (rectangle). Black arrows represent primers specific to the L1 sequence. Combinations of these primers are used to generate the following amplicons (arranged top to bottom): 5′ L1-genome junction, 3′ L1-genome junction, L1 insertion (filled site), and empty site. (C) PCR validation results for a *de novo* L1 insertion detected in cell line hiPSC-CRL2429. An empty/filled PCR was also performed with cell line hiPSC-CRL1502 as a negative control. Red and black arrow heads indicate the expected filled and empty site band sizes, respectively. NTC, nontemplate control. (D) *De novo* L1 insertion sequence structure. In addition to TSDs (triangles), the full-length L1-Ta insertion was flanked by 5′ (orange) and 3′ transductions (purple). (E) The same experiments as described for panel C except that they were performed for the donor L1 responsible for the *de novo* L1 insertion (left) and its lineage progenitor L1 (right), using CRL2429 fibroblast genomic DNA.

To study endogenous retrotransposition during neurogenesis, we obtained two hiPSC lines (hiPSC-CRL1502 and hiPSC-CRL2429) generated via delivery of defined reprogramming factors to healthy human dermal fibroblasts (58, 76). We then differentiated each hiPSC line toward a neuronal phenotype for 156 days in culture (Fig. 1A) and applied retrotransposon capture sequencing (RC-seq) (58, 69, 77) to genomic DNA sampled from the parental fibroblasts (time point 0 [*T*_0_]), hiPSCs (*T*_1_), and several time points of differentiation (*T*_2_ to *T*_6_) (Table 1). Two earlier passages of each hiPSC line were also analyzed by RC-seq to better distinguish L1 insertions arising during reprogramming or cell cultivation (Table 1). Cells from each point of neurodifferentiation were characterized by immunocytochemistry (Fig. 1A) and included neural epithelium (*T*_2_), neural rosettes denoting immature neurons (*T*_3_) and three stages of prolonged neuronal maturation (*T*_4_ to *T*_6_). Endogenous L1 insertions detected by RC-seq and absent from the reference genome were annotated as either polymorphic (previously published or present at *T*_0_) or *de novo* (only present at *T*_1_ or later in one time course). Two potential *de novo* L1 insertions were identified (see Table S1 in the supplemental material). We then performed insertion site-specific PCR validation for each event (Fig. 1B and Table 2) and found that one insertion, on chromosome 1 (Chr1), was *de novo* in hiPSC-CRL2429 cells at time point *T*_1_*_,_* was carried through neurodifferentiation (Fig. 1C), and was absent from hiPSC-CRL1502 (Fig. 1C). PCR indicated that the other putative *de novo* event was polymorphic because it was found in the matched parental fibroblast population (Table 2 and Table S1).

**TABLE 1:** RC-seq library information

hiPSC line and library DNA input^*c*^	Time point	RC-seq reads^*b*^		RC-seq data source
		Count	Aligned %	
CRL1502				
Fibroblasts	*T*_0_	44,033,582	99.95	Klawitter et al.
hiPSCs p76^*a*^	*T*_1_	42,151,994	99.74	This study
Neural epithelium	*T*_2_	33,972,001	99.77	This study
Immature neurons	*T*_3_	39,766,940	99.77	This study
Neurons I	*T*_4_	47,155,514	99.78	This study
Neurons II	*T*_5_	44,381,111	97.91	This study
Neurons III	*T*_6_	36,222,610	99.77	This study
hiPSCs p15	Earlier hiPSC passage	24,385,022	99.88	Klawitter et al.
hiPSCs p40	Earlier hiPSC passage	63,130,772	99.88	Klawitter et al.
CRL2429				
Fibroblasts	*T*_0_	24,386,590	99.91	Klawitter et al.
hiPSCs p70	*T*_1_	38,460,241	99.63	This study
Neural epithelium	*T*_2_	40,174,554	99.79	This study
Immature neurons	*T*_3_	46,646,999	99.78	This study
Neurons I	*T*_4_	27,279,492	99.79	This study
Neurons II	*T*_5_	46,018,310	99.77	This study
Neurons III	*T*_6_	36,033,944	99.54	This study
hiPSCs p11	Earlier hiPSC passage	64,534,189	99.40	Klawitter et al.
hiPSCs p40	Earlier hiPSC passage	27,447,967	99.39	Klawitter et al.

a p, passage.

b Data from 2- by 150-mer reads.

c Neurons I, II, and III were harvested after 72, 112, and 156 days of differentiation *in vitro*, respectively.

**TABLE 2:** PCR primers used for validation and bisulfite sequencing

Primer function and name	Sequence
Genomic primers for empty/filled L1 validation reactions	
LineageProgenitor_Chr11_fwd	AGGAAACAGTGAGGGGAAGC
LineageProgenitor_Chr11_rev	TGAGGCCCAGGAGTCATATC
Donor_Chr3_fwd	TGTATGACAGTAAAATAATGGGTAGATGA
Donor_Chr3_rev	CTGGCCTCTTCACTGCATTT
DeNovo_Chr1_fwd	CTGGTAACCCCAGAATGACG
DeNovo_Chr1_rev	ATCCTGCCTCAGCGAACTTA
Non-ref_Chr3_fwd	TTGTGGGAAGGCAAAATGAT
Non-ref_Chr3_rev	TATTCAATCCCAACCCAGGA
L1-specific primers for validation of 5′ and 3′ L1-genome junctions	
hL1_273_rev	ACCCGATTTTCCAGGTGCGT
hL1_ACshort_fwd	AGATATACCTAATGCTAGATGACAC
NotI/L1-genome junction-spanning primers for cloning full-length L1s	
LineageProgenitor_Chr11_NotI_fwd	CAAGCGGCCGCTTACATTTTTAAAGAATTGTAGGGGAG
Donor_Chr3_NotI_fwd	TAAAGCGGCCGCAACAGAATGAGTAAATAATGGAGGG
DeNovo_Chr1_NotI_fwd	TTCGCGGCCGCATTAAAGAAATGACATCTGAAATAATGGA
Non-ref_Chr3_NotI_fwd	CAACGCGGCCGCTTAAAGTTAAAGACACGG
L1-specific primers for sequencing full-length L1s	
L1_452_fwd	GCCCAGGCTTGCTTAGGTA
L1_1020_fwd	TGATTTTGACGAGCTGAGAGAA
L1_1532_fwd	CCTCGAGAAGAGCAACTCCA
L1_1966_fwd	GCAAAATCACCAGCTAACATCA
L1_2494_fwd	AACTCAGCTCTGCACCAAGC
L1_3014_fwd	AAATCAGAGCAGAACTGAAGGAAA
L1_3502_fwd	GAGGCCAGCATCATTCTGATA
L1_4022_fwd	CAATCAGGCAGGAGAAGGAA
L1_4472_fwd	TCCCCATCAAGCTACCAATG
L1_4973_fwd	TGTCCAAAACACCAAAAGCA
L1_5492_fwd	TACCATTTGACCCAGCCATC
Primers for amplification of L1 promoters from bisulfite converted DNA	
L1_Bis-LP	GATTTGTTTTTGGATTGTAAAATGGTT
L1_Bis-Donor	TGGGTAGATGAACAGATAAGTAAA
L1_BiS-DN	GTTATTTGATAGTATTTTAATGAAGATT
L1_Bis-F	TAGGGAGTGTTAGATAGTGG
L1_Bis-R	ACTATAATAAACTCCACCCAAT

We then cloned and capillary sequenced the entire *de novo* L1 insertion (Fig. 1D) and manually inspected the integration site for hallmarks of TPRT (8, 9, 16, 17). The L1 was full length, belonged to the L1-Ta subfamily, carried 5ʹ and 3ʹ transductions, was flanked by 16-nucleotide (nt) TSDs, inserted at a degenerate L1 endonuclease motif (5ʹ-TT/AAAG), and terminated with a 33-nt poly(A) tract. The 5ʹ and 3ʹ transductions were 10 nt and 44 nt in length, respectively, and the 3ʹ transduction was preceded by an internal 17-nt poly(A) tract (Fig. 1D). These features were consistent with endogenous retrotransposition mediated via TPRT and, as confirmed by insertion site-specific PCR, showed that the *de novo* L1 insertion represented a bona fide retrotransposition event occurring during reprogramming, or very early in hiPSC-CRL2429 cultivation.

### An extended human RC-L1 transduction family.

The *de novo* L1 insertion was the first such example to be found in hiPSCs of an endogenous L1 insertion carrying both 5ʹ and 3ʹ transductions. These transductions uniquely indicated a donor L1 sequence on chromosome 3 that was heterozygous in the hiPSC-CRL2429 parental fibroblast population (Fig. 1E). The donor L1 was absent from the reference genome and was polymorphic in humans; it was previously shown to mobilize efficiently *in vitro* (35). To identify any other germ line L1 insertions closely related to the donor L1, we aligned the 3ʹ transduced sequence to the reference genome and to the annotated 3ʹ L1-genome junction sequences of polymorphic L1s carried by hiPSC-CRL2429 or hiPSC-CRL1502 (Table S1) or those annotated by previous studies (52, 58, 69, 70, 77, 78). We further annotated this list with results obtained by previous studies of L1 mobilization in the germ line, tumors, and cancer cell lines (3, 35, 37, 49, 77, 79–87). From this analysis, we reconstructed an extended L1 transduction family comprising 14 members (Table 3), including a plausible founder, or lineage progenitor (44), element for the family, which was homozygous in hiPSC-CRL2429 and located on chromosome 11 (Fig. 1E).

**TABLE 3:** Transduction family members

Element	Genomic coordinate (hg19)	TSD	Full-length^*a*^	Identification source and/or reference(s)
Lineage progenitor L1	Chr11: 95169381	AAAGAATTGTA	Y	Reference genome; 3
Donor L1	Chr3: 38626082	AGAATGAGTAAATAATG	Y	35, 49, 71, 77, 79, 82–87
*De novo* L1	Chr1: 231719316	AAAGAAATGACATCTG	Y	This study
Ref_Chr7_q21.3	Chr7: 96475963	GAAAGTTCCAGTTGC	Y	Reference genome
Non-ref_Chr3_p24.3	Chr3: 20748904	TAAAGACAC	Y	35, 49, 71, 77, 79, 82, 83, 87
Ref_Chr1_p31.1_a	Chr1: 84518060	AGAAAAACAAATCA	Y	Reference genome
Ref_Chr1_p31.1_b	Chr1: 83125969	AAAAAAAATGGTTCATGC	N	Reference genome
Ref_Chr9_p23	Chr9: 12556931	GAAAAGTATTGTATTG	N	Reference genome
Non-ref_Chr3_p12.2_a	Chr3: 80590176	GAAAATGGAATGGG	Y	35, 37, 49, 77, 79, 82, 83
Non-ref_Chr3_p12.2_b	Chr3: 82144869	AGAAATAATAATTTCC	Y	49, 71, 77, 79, 83, 85, 86
Non-ref_ChrX_p11.4	ChrX: 38097551	AAAAGCGATATG	Y	49, 86
Non-ref_Chr17_q12	Chr17: 32813609	AAGAAGGTAAGATGG	N	71, 77, 79, 82–84, 87
Non-ref_Chr1_p22.2	Chr1: 90914512	AAAAAGCTCTTTCAG	N	49, 71, 77, 79, 85, 86
Non-ref_Chr4_q12	Chr4: 53628490	TAAATTACAGGTTA	N	49, 71, 77, 79, 82, 83, 86

a Y, yes; N, no.

**FIG 2:**
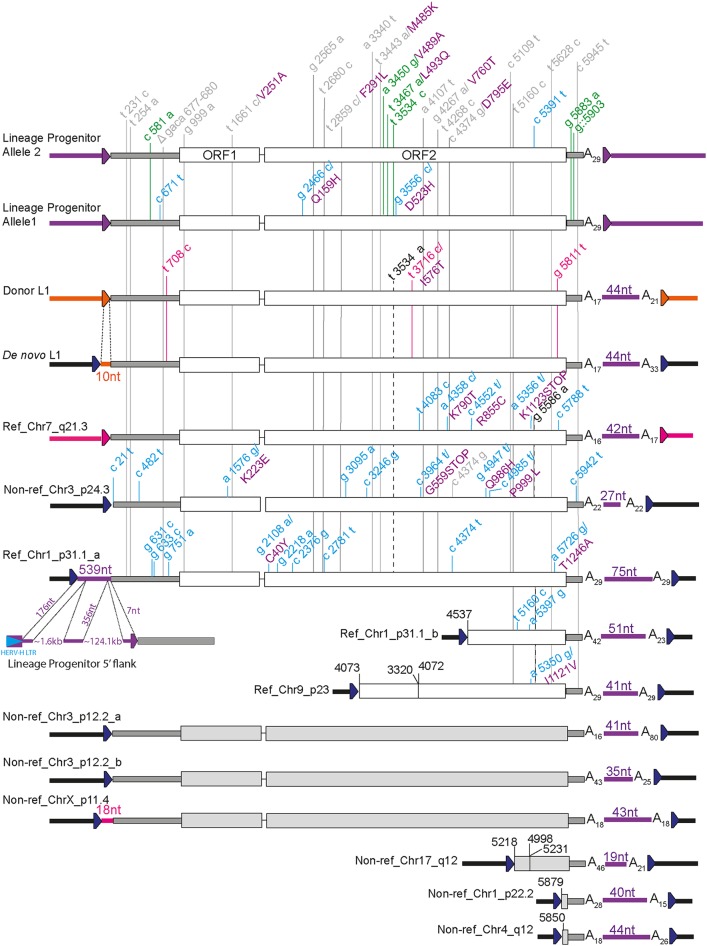
The reprogramming-associated *de novo* L1 insertion belonged to an extended L1 transduction family. The diagram shows 14 members of this family, including the *de novo* L1 insertion. Two alleles of the lineage progenitor L1 were characterized. TSDs flanking each L1 are represented by blue arrows. The 5ʹ UTR and 3ʹ UTR sequences are shown in dark gray, while ORFs with known and unknown sequences are shown in white and light gray, respectively. Transduction colors match their source L1 locus: donor L1 → *de novo* L1 (orange), Ref_Chr7_q21.3 → Non-ref_ChrX_p11.4 (pink), lineage progenitor L1 → all other family members (purple). Letter and number combinations within L1s correspond to L1.3 nucleotide (lowercase) and ORF1 and ORF2 amino acid (purple uppercase) positions (88). Nucleotide changes versus L1.3 and present in all, some, or one of the sequenced members of the transduction family are shown in gray, black, and blue, respectively. Nucleotide changes unique to the two alleles of the lineage progenitor are shown in green, and nucleotide changes unique to the donor L1 and *de novo* L1 are shown in pink.

To further characterize the transduction family, we analyzed the complete internal sequence of eight of its members found in either hiPSC-CRL1502 or hiPSC-CRL2429, including the *de novo* L1 insertion. A consensus sequence was obtained for the lineage progenitor, donor, and *de novo* L1s, as well as for another L1 nonreference (Non-ref) element, named Non-ref_Chr3_p24.3, via capillary sequencing of multiple full-length amplicons derived from independent PCRs (Fig. 2). Internal and flanking sequences for four additional reference (Ref) elements (Ref_Chr7_q21.3, Ref_Chr1_p31.1a, Ref_Chr1_p31.1b, and Ref_Chr9_p23) were obtained from the reference genome assembly. The 5′ and 3′ L1-genome junctions of the remaining six nonreference elements (Non-ref_Chr3_p12.2_a, Non-ref_Chr3_p12.2_b, Non-ref_ChrX_p11.4, Non-ref_Chr17_q12, Non-ref_Chr1_p22.2, and Non-ref_Chr4_q12) were provided by previous studies (Table 3).

**FIG 3:**
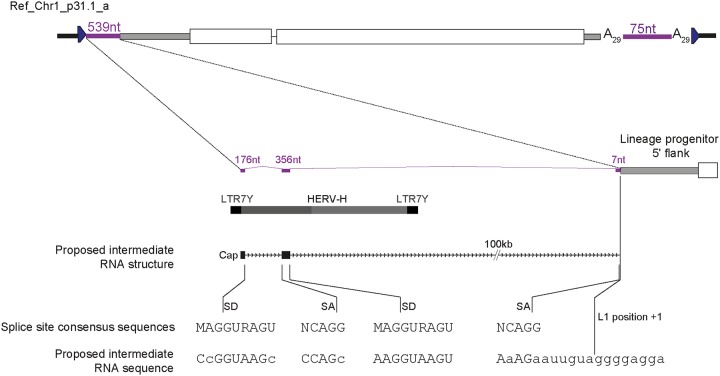
A spliced RNA initiated from an HERVH LTR7Y transcription start site upstream of the lineage progenitor L1 provided the proposed intermediate template for the 5′ transduction carried by Ref_Chr1_p31.1a. The proposed RNA structure and splice donor (SD) and acceptor (SA) sequences are provided, as are the consensus splice donor and acceptor motifs. An untemplated guanine was present at the 5′ end of Ref_Chr1_p31.1a, suggesting that the proposed intermediate template RNA was capped (18, 89).

Notably, the homozygous lineage progenitor L1 had two allelic variants in hiPSC-CRL2429 cells, which were distinguished by four single nucleotide variants. Allele 1 contained a nonsynonymous change (D523H) in the ORF2p RT domain, which was not found in allele 2. Further analysis of the remaining family members relative to the sequence of L1.3 (88) indicated that each contained internal single nucleotide variants common to both progenitor element alleles, in addition to shared 3ʹ transduced sequences (Fig. 2). The *de novo* and donor elements were identical in their L1 sequences, and the 5ʹ transduced sequence carried by the *de novo* insertion exactly matched the 10 nt directly upstream of the donor element. Surprisingly, in addition to the *de novo* L1 insertion, two other elements, Ref_Chr1_p31.1_a and Non-ref_ChrX_p11.4, each carried both 5ʹ and 3ʹ transductions, enabling us to unambiguously identify their respective donor L1 sequences (the lineage progenitor and Ref_Chr7_q21.3, respectively), which were also members of the transduction family (Fig. 2). Interestingly, the 539-nt 5ʹ transduction carried by Ref_Chr1_p31.1a was preceded by a single untemplated guanine, suggesting that the template mRNA was capped (18, 89), and utilized a transcription start site in the 5ʹ long terminal repeat (LTR7Y) sequence of a human endogenous retrovirus type H (HERV-H) provirus integrated ∼126 kb upstream of the lineage progenitor L1 (Fig. 2). This mRNA template incorporated two exons upstream of the lineage progenitor L1, which were spliced together and to the L1 via sites strongly resembling consensus mammalian splice donor and acceptor sequences (Fig. 3). Another element, Non-ref_Chr3_p24.3, incorporated a nonsense mutation predicted to truncate ORF2 prior to its RT domain. In sum, these experiments characterized relationships among members of a transduction family, which, in many cases, remain potentially capable of retrotransposition in the germ line, in tumors (37, 49), and, as shown here, in hiPSCs.

### Transduction family mobilization *in vitro*.

**FIG 4:**
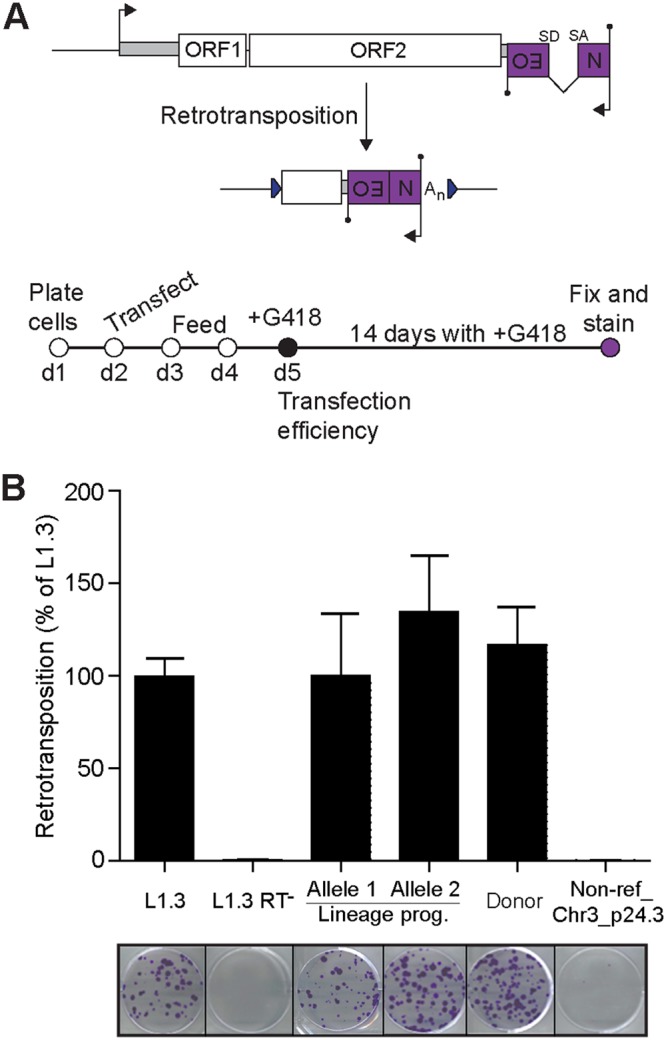
L1 transduction family members are retrotransposition competent *in vitro*. (A) At top is a schematic of the cultured-cell L1 retrotransposition reporter assay (8). An L1 driven by its native promoter is tagged with an intron-containing (SD, splice donor; SA, splice acceptor) G418 antibiotic resistance gene cassette (Neo) oriented antisense to the L1 (black circle, polyadenylation signal). Transcription, splicing, and retrotransposition of the L1 reporter generates newly integrated engineered L1 insertions with a functional and expressed Neo cassette. The bottom diagram is a summary of the retrotransposition assay protocol. (B) L1 transduction family members were tested via the L1 retrotransposition reporter assay in cultivated HeLa cells. Elements included positive (L1.3) (88, 91) and negative (L1.3 RT^−^) (6) controls, two identified alleles of the lineage progenitor L1, the donor L1 (identical in sequence to the *de novo* L1), and Non-Ref_Chr3_p24.3, which encoded an ORF2 stop codon. The assay was repeated three times (biological replicates) with similar results. Values represent the means ± standard deviations of colonies counted in each of three technical replicates, normalized to the value for L1.3. Representative images matching each element, tested in six-well plates, are shown below.

To assess the retrotransposition competence of several members of the transduction family, we employed a cultured-cell-engineered L1 retrotransposition reporter assay (8) in HeLa cells. Briefly, in this assay, an L1 sequence is cloned into a vector containing an antibiotic resistance cassette oriented antisense to the L1 copy, where the resistance gene contains an intron oriented in sense to the L1, meaning antibiotic resistance occurs only after splicing and retrotransposition of the reporter cassette (8, 90) (Fig. 4A). Through this approach, we tested the following elements: a known hot RC-L1 (L1.3) as a positive control (88, 91), an RT mutant L1 (L1.3 RT^−^) as a negative control (6), both detected alleles of the lineage progenitor L1, the donor L1 (identical in sequence to the *de novo* L1), and Non-ref_Chr3_p24.3, which contained an ORF2 stop codon in its RT domain (Fig. 2). Each element was tested in triplicate experiments under the control of its native L1 promoter (Fig. 4B).

Among the tested elements, the lineage progenitor L1 allele 2 exhibited the highest retrotransposition frequency activity, at 135% of L1.3 (Fig. 4B). Consistent with the progenitor L1 allele 1 carrying two nonsynonymous mutations in ORF2 not found in allele 2, resulting in Q159H and D523H amino acid changes (Fig. 2), we found allele 1 retrotransposed at ∼74% of the efficiency observed for allele 2 and at a similar efficiency as seen for L1.3 (Fig. 4B). Each progenitor L1 allele jumped at >10% of the efficiency of L1.3 and therefore met the definition of a hot RC-L1 (35). Notably, an allele of the progenitor L1 had previously been tested, albeit in an osteosarcoma cell line and with a different reporter system, and was found to present much more limited mobilization potential *in vitro* (3). The most likely explanation for this difference is that the prior study tested an allele of the progenitor L1 not assayed here. This result further highlights the impact of allelic variation upon the retrotransposition efficiency of a given genomic RC-L1 copy (38, 39).

The donor L1 was sequenced from a line (hiPSC-CRL2429) established from a Caucasian individual. Apart from a single nucleotide mutation in its 3ʹ UTR, this L1 was identical to one identified in a Japanese individual by a previous study, which reported its retrotransposition efficiency as 101% of L1.3 in the same reporter assay (35). Here, the donor L1 jumped at 117% of L1.3, corroborating the prior experimental results and confirming that retrotransposition-competent alleles of this L1 exist in multiple human populations. Finally, L1.3 RT^−^ and Non-ref_Chr3_p24.3 did not retrotranspose, consistent with disabled ORF2 RT activity in each case (Fig. 4B). Overall, these results demonstrate that the *de novo* L1, its donor sequence, and the progenitor element of the transduction family were all hot RC-L1s *in vitro*.

### L1 promoter methylation is dynamic during neurodifferentiation.

**FIG 5:**
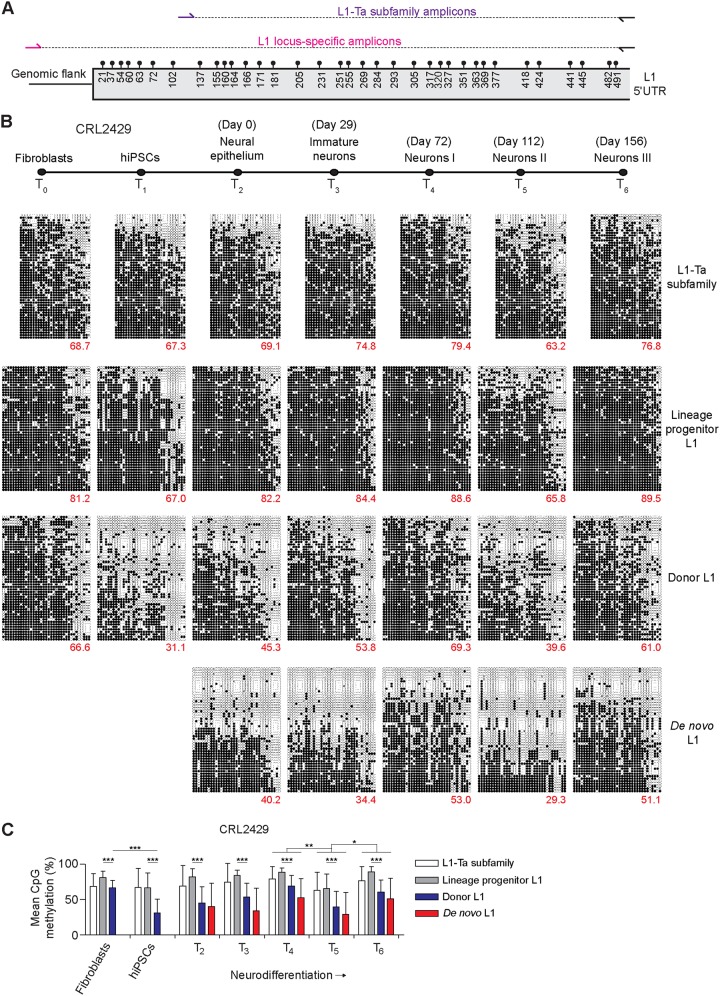
L1 promoter DNA methylation is dynamic during hiPSC-CRL2429 reprogramming and neurodifferentiation. (A) L1 bisulfite sequencing analysis design. CpG dinucleotides are indicated by circles above the L1 5ʹ UTR, and their nucleotide positions are provided below. A common reverse primer (black) is combined with either an L1-Ta subfamily forward primer (purple) or an L1 locus-specific forward primer (pink) to generate PCR amplicons for multiplexed paired-end Illumina 2- by 300-mer sequencing, resolving each amplicon in full. (B) L1 CpG methylation patterns in hiPSC-CRL2429 fibroblasts, hiPSCs, and neural cells derived *in vitro*. Each cartoon panel corresponds to an amplicon (L1-Ta subfamily or specific L1 locus) and displays 50 random, nonidentical sequences (black circle, methylated CpG; white circle, unmethylated CpG; ×, mutated CpG). The percentage of methylated CpG is indicated in the lower right corner of each cartoon. (C) L1 promoter CpG methylation levels for the hiPSC-CRL2429 neurodifferentiation time course. Values represent the means ± standard deviations of CpG methylation of the corresponding 50 reads for each amplicon, as presented in panel B. Statistical analyses involved paired *t* tests, with a Bonferroni multiple-testing correction where appropriate. *, P < 0.01; **, P < 0.001; ***, P < 0.0001.

**FIG 6:**
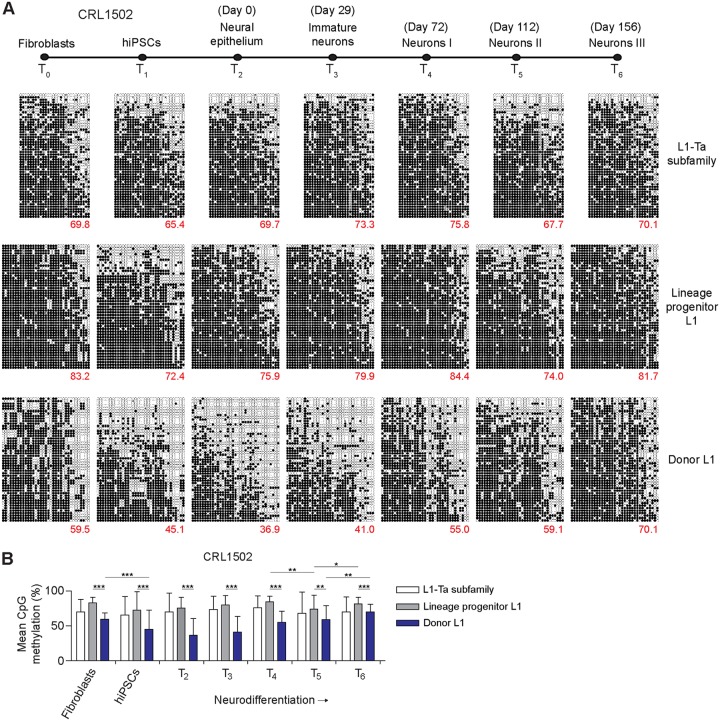
L1 CpG methylation patterns in hiPSC-CRL1502 fibroblasts, hiPSCs, and neural cells derived *in vitro*. (A) Each cartoon panel corresponds to an amplicon (L1-Ta subfamily or specific L1 locus) and displays 50 random, nonidentical sequences (black circle, methylated CpG; white circle, unmethylated CpG; ×, mutated CpG). The percentage of methylated CpG is indicated in the lower right corner of each cartoon. (B) L1 promoter CpG methylation levels for the hiPSC-CRL1502 neurodifferentiation time course. Values represent the means ± standard deviations of CpG methylation of the corresponding 50 reads for each amplicon, as presented in panel A. Statistical analyses involved paired *t* tests, with a Bonferroni multiple-testing correction where appropriate. *, P < 0.01; **, P < 0.001; ***, P < 0.0001.

Full-length L1 mRNA transcription is a prerequisite for L1 retrotransposition in *cis* and is directed by an internal promoter located in the L1 5ʹ UTR (25). DNA methylation of an adjacent CpG island mediates repression of the L1 promoter (26, 31). Genome-wide, the L1-Ta subfamily is thought to be broadly hypomethylated in pluripotent cells and then methylated during differentiation, including in mature neurons (40, 49, 58, 61, 63, 67). However, the temporal methylation patterns for the L1-Ta subfamily and individual L1-Ta promoters during the various stages of neurodifferentiation to date have not been resolved. It is also unknown how quickly methylation is established upon new L1 insertions that arise in pluripotent cells. To address these questions, we applied a multiplexed L1 locus-specific bisulfite sequencing approach (52, 78) (Fig. 5A and Table 2) to assess DNA methylation among the *de novo*, donor, and progenitor L1 5ʹ UTR sequences, as well as the L1-Ta subfamily genome wide. This analysis was performed for both hiPSC lines and their parental fibroblasts and derivative neuronal cell populations, as surveyed by RC-seq, with the exception of the *de novo* L1, which was present only in hiPSC-CRL2429 (Fig. 5B and 6).

Considering general trends observed in both hiPSC lines, the L1-Ta subfamily and individual L1 promoters were most methylated in fibroblasts and differentiated neurons and least methylated in hiPSCs and the earliest stages of neurodifferentiation (Fig. 5B and 6A). For example, 66.6%, 31.1%, and 61.0% of CpG dinucleotides surveyed in the donor L1 were methylated, on average, in hiPSC-CRL2429 fibroblasts, hiPSCs, and mature neurons, respectively. Among the two hiPSC lines, the highly significant (*P* < 0.0001, paired *t* test with Bonferroni correction) reductions in methylation observed for the donor L1 during hiPSC derivation (25.0% on average) far exceeded that seen for the lineage progenitor (12.5%) and L1-Ta subfamily (2.9%) (Fig. 5C and 6B). The lineage progenitor L1 was significantly (*P* < 0.001, paired *t* test) more methylated than the donor L1 at all time points in each hiPSC line, with the L1-Ta subfamily being methylated to a level between that of the lineage progenitor L1 and donor L1 at most time points (Fig. 5C and 6B). Notably, we observed a significant (*P* < 0.001, paired *t* test with Bonferroni correction) reduction in methylation (23.1% average decrease) for all amplicons at *T*_5_ in hiPSC-CRL2429, followed by a significant (*P* < 0.01) increase in methylation at *T*_6_ (20.1% average increase) (Fig. 5C). This trend was also observed at *T*_5_ for hiPSC-CRL1502, except for the donor L1 (Fig. 6B). The reasons for this pattern are presently unclear (see Discussion). Overall, these results demonstrate that DNA methylation is far more dynamic during reprogramming and differentiation for a donor L1 that can mobilize during or shortly after reprogramming than is seen for the vast majority of L1-Ta subfamily elements.

The *de novo* L1, which arose in hiPSC-CRL2429, could be detected at its 5ʹ L1-genome junction by site-specific PCR at time points *T*_1_ through *T*_6_ (Fig. 1C). However, as assessed by the number of unique sequencing reads generated, the PCR amplicon pool for the *de novo* L1 was very low in complexity at *T*_1_, perhaps due to a low percentage of cells carrying the mutation, and we therefore excluded *T*_1_ from further analysis. The *de novo* L1 was nonetheless consistently less methylated than its donor L1 in hiPSC-CRL2429 time points *T*_2_ through *T*_6_, with average values across these stages of 41.6% and 53.8%, respectively (Fig. 5B). Methylation ultimately increased upon the *de novo* L1 during neurodifferentiation, but even in neurons we observed a significant number of cells in which the *de novo* L1 promoter was fully demethylated. For the donor L1 and the L1-Ta subfamily, we also observed instances of cells in which these promoters were fully demethylated at various points of neuronal differentiation (Fig. 5B, Fig. 6A). These results suggest that the *de novo* L1 was only partially methylated subsequent to its integration into the hiPSC-CRL2429 genome and remained incompletely methylated in mature neurons.

Given the disparate methylation levels observed for the *de novo* and donor L1 promoter regions compared to the level of the lineage progenitor L1, we examined predicted DNA-binding protein motifs (92) affected by sequence variation among these elements (Fig. 2). The 10-nt 5′ transduction carried by the *de novo* L1 insertion incorporated a perfect FOX (forkhead box) protein binding motif (93). Members of the FOX protein family can act as “pioneer” factors in the developmental activation of promoters located in heterochromatin (94). In addition, the T708C nucleotide mutation present in the *de novo* and donor L1 copies greatly increased the predicted binding affinity for retinoid X receptor (RXR) proteins to this site. RXR proteins are known to respond to vitamin A (95), which is a component of the B-27 medium used here for neurodifferentiation. Conversely, the C581A nucleotide mutation carried by the lineage progenitor L1, and not by the *de novo* or donor L1 sequences or any other member of the transduction family, removed a key nucleotide mismatch from the core of a predicted PU.1 binding motif. PU.1 is established to recruit DNA methyltransferases to genomic loci and to form a repressor complex with MeCP2, which is a key mediator of L1 silencing (96–98). These *in silico* analyses suggested that differential DNA-binding protein activity as a result of sequence variation may impact the methylation and transcriptional state of members of the transduction family.

## DISCUSSION

The L1 transduction family identified here is the largest found to date and adds to other such families characterized by previous studies (35, 44, 54). Although the extent of the transduction family is revealed here, it is likely that additional members will be identified in the future. It should also be noted that each transduction family member, aside from the *de novo* L1, was either present in the reference genome or identified by earlier works (Table 3). Unusually, in addition to 3ʹ transduced sequences, 3 of the 14 family members carried 5ʹ transductions. This 5ʹ transduction frequency (21.4%) is exceptionally high, given how rarely such events are found in the human germ line (1). Two of the 5ʹ transductions were relatively short (10 nt, *de novo* L1; 18 nt, Non-ref_ChrX_p11.4) and likely resulted from the L1 promoter directing mRNA transcriptional initiation upstream of L1 position +1. The third 5ʹ transduction identified was significantly longer (539 nt, Ref_Chr1_p31.1_a) and resulted from transcription initiated by the 5ʹ LTR of an upstream HERV-H proviral sequence, followed by splicing of this mRNA into a site adjacent to the donor L1. The inclusion of both LTR and internal HERV-H sequences in an L1 5ʹ transduction was an intriguing result as most heritable L1 insertions appear to arise early in mammalian embryogenesis (55, 56), and HERV-H elements are highly expressed in pluripotent cells (99–103). To speculate, this example demonstrates how HERV-H activation in the early embryo could lead to L1 mobilization. Nonetheless, it remains unclear why 5ʹ transductions are generally so frequent in this family and not in other transduction families (35, 44, 54). One possibility, an ORF2p amino acid change supporting elevated RT processivity and therefore increased average L1 insertion length, was excluded by an inspection of nonsynonymous sequence variants in this region (Fig. 2). Also excluded was the more likely possibility of mutations in known YY1, RUNX3, or SOX transcription factor binding sites (41, 104, 105) in the lineage progenitor L1 5ʹUTR or in alternative predicted sites located in the immediate 100 nt of its 5′ genomic flank, which may alter the accuracy of RNA polymerase II transcriptional initiation (Fig. 2). Otherwise, the family exhibited extensive variation in 3ʹ transduction and poly(A) tail length, as reported elsewhere for L1 insertions arising from a common donor L1 in the human population and cancer genomes (32, 37, 44, 49, 52, 78).

The discovery of a *de novo* L1 insertion in hiPSC-CRL2429 corroborates previous reports of endogenous and engineered L1 retrotransposition associated with reprogramming and hiPSC cultivation (58, 61). L1-mediated mutagenesis is potentially an important consideration for the use of hiPSCs in biomedical applications and as models of disease because the phenotypic properties of hiPSCs and their cellular derivatives could be compromised as a result of *de novo* L1 insertions (58, 106). We demonstrate here that an endogenous L1 insertion arising in an hiPSC line is maintained during neurodifferentiation, indicating that such events can be present in differentiated cell lines derived from hiPSCs. In this case, the L1 was intergenic, and the accompanying transductions did not include protein-coding exons or regulatory elements (47), lessening the probability of a functional impact in neurons carrying the L1 insertion. Although endogenous L1 retrotransposition is established to occur in the neuronal lineage (65), we did not identify any additional *de novo* L1 insertions that were restricted to neural cells. These events were likely to each be carried by very few cells, meaning that they may not accrue sufficient RC-seq read depth to meet the detection thresholds used here. Nonetheless, it is plausible that *de novo* L1 insertions that impact the phenotype of hiPSC-derived cells will be identified in the future, especially as gene expression changes have been observed coincident with intronic L1 insertions arising during hiPSC generation (58).

DNA methylation is thought to be established on L1 sequences very early in mammalian embryogenesis (27, 28, 58, 61, 63, 67) and maintained in mature neurons. To our knowledge, L1 promoter methylation has not been explored for the various multipotent and immature neuronal cell types that arise during neurogenesis. Using *in vitro* hiPSC neurodifferentiation to represent neuronal development and maturation *in vivo*, we found that L1 promoter methylation was highly dynamic and increased as neurons matured. In each hiPSC line studied, we observed cells at multiple stages of neurodifferentiation, including mature neurons, where the donor L1 and other L1-Ta promoters were fully demethylated. Although the donor L1 was demethylated in hiPSCs compared to the methylation level of the matching parental fibroblasts, the absolute magnitudes of this change were dissimilar in the two lines (35.5% and 14.4% for hiPSC-CRL2429 and hiPSC-CRL1502, respectively). This perhaps reflected natural variation in the cohort of RC-L1s hypomethylated in each individual, before and after reprogramming. At time point *T*_5_, which follows a gliogenic switch (107–109) during neural differentiation, we also observed a consistent reduction in L1 promoter methylation. This phenomenon could reflect a genome-wide reduction in DNA methylation specific to this stage of neurodifferentiation, perhaps due to a shift in the proportion of glial and neuronal cells present in culture, and warrants further study.

The *de novo* L1 insertion appeared to be rapidly targeted for repression by the host genome. During neurodifferentiation, similar transitions in methylation were observed for the *de novo*, donor and lineage progenitor L1s, and the L1-Ta subfamily even if the absolute methylation levels were very different among these elements. This result was consistent with epigenomic remodeling during reprogramming and neurodifferentiation (110, 111) impacting the ground state of L1 methylation genome-wide. It also suggested that the *de novo* L1 insertion was quickly identified and regulated by the same pathways acting upon extant L1 copies on the genome even if the degree of methylation upon the *de novo* L1 was significantly lower than that applied to the transduction family and its ancestral L1-Ta subfamily. L1 5' UTR sequence variants, for example the C581A nucleotide mutation carried by the lineage progenitor L1 and predicted to increase DNA methylation mediated by PU.1, could contribute to differential methylation patterns among members of the transduction family. It is also notable that the *de novo* L1 remained retrotransposition competent, as do many other L1 insertions occurring in hiPSCs or arising during human embryogenesis (57, 58). To speculate, if hiPSCs are taken as a model of very early development, a milieu where most heritable L1 insertions arise (55), it is plausible that RC-L1 insertions arising *de novo* in this context will be incompletely methylated during later development and therefore possess a disproportionate capacity for further mobilization in the soma. Ultimately, hiPSCs and hESCs present accessible models to predict how L1 subfamilies and individual L1 loci are regulated. Additional work is required to test whether these patterns are observed during mammalian development *in vivo*.

## MATERIALS AND METHODS

### hiPSC generation and neuronal differentiation.

Human induced pluripotent stem cell lines were episomally derived as previously described (76). Neuronal differentiation was performed as described previously (112) with slight modifications. Prior to neuronal differentiation, feeder-free hiPSCs were cultured in murine embryonic fibroblast (MEF)-conditioned KOSR medium supplemented with 100 ng/ml basic fibroblast growth factor (b-FGF). Initiation of neuronal differentiation occurred with the supplementation of dual SMAD inhibitors SB431542 (10 μM) and dorsomorphin (1 μM) into knockout serum replacement (KOSR) medium, which was gradually exchanged for 3 N medium (1:1 medium mix of N-2- and B-27-containing medium comprised of 1:1 neurobasal/Dulbecco’s modified Eagle’s medium [DMEM]–F-12 supplemented with 2% B-27, 1% N-2, 2 mM GlutaMax, 2.5μg/ml insulin, 0.05mM nonessential amino acids [NEAA], 0.05 mM beta-mercaptoethanol [all from Life Technologies]) in 25% incremental steps on days 4, 6, 8, and 10. Neural rosettes were selectively harvested and plated on Matrigel-coated TC dishes and expanded in 3 N medium supplemented with 20 ng/ml b-FGF. Around day 30 early neuronal progenitors were harvested with Accutase and seeded onto poly-l-ornithine/laminin-coated dishes (0.01% weight/volume and 20 μg/ml, respectively), and maintained in 3 N medium for the remainder of neurodifferentiation.

### Immunocytochemistry.

Neural cultures were grown on Matrigel-coated plastic coverslips in 3 N medium and were fixed in 4% paraformaldehyde (Sigma) in phosphate-buffered saline (PBS) for 15 min at room temperature and permeabilized in 0.01% Triton X-100 (Ajax Finechem) in PBS for 15 min at room temperature. All cells were blocked for 1 h with 10% goat serum (Invitrogen) in PBS. Primary antibodies used were OCT4 (1:100; Millipore), NANOG (1:100; Millipore), CUX1 (1:100; Abcam), glial fibrillary acidic protein (GFAP) (1:250; Dako), TUBB3/TUJ1 (1:1,000; Covance), BRN2 (1:100; Abcam), PAX6 (1:1,000; Developmental Studies Hybridoma Bank [DSHB]), anti-phospho-histone H3 (Ser10) (1:200; Cell Signaling Technology) and were applied for 3 to 4 h at room temperature or overnight at 4°C. Isotype- and species-matched Alexa Fluor-conjugated secondary antibodies (1:1,000; Invitrogen) were applied for 1 h at room temperature. Cells were washed in PBS and mounted on glass slides with ProLong Gold antifade containing 4′,6′-diamidino-2-phenylindole (DAPI; Invitrogen) and imaged using an Olympus IX51 (Olympus) fluorescence microscope equipped with a MicroPublisher, version 3.3, real-time viewing (RTV) charge-coupled-device (CCD) camera (QImaging) using Q-Capture Pro, version 6.0, software.

### Nucleic acid extraction.

A total of approximately 500,000 cells per time point were pelleted (1,000 rpm for 5 min) and then washed with Dulbecco’s phosphate-buffered saline (DPBS) (14190144; Gibco) and pelleted again (1,000 rpm for 5 min) and resuspended in 100 μl of UltraPure DNase/RNase-free distilled water (10977023; Gibco). Cells were lysed in 10 mM Tris, pH 9.0, and 1 mM EDTA, with 2% SDS and 100 μg/ml proteinase K at 65°C. A final concentration of 10 μg/ml RNase A was added to each sample and incubated at 37°C for 30 min. DNA was extracted using phenol-chloroform-isoamyl alcohol (25:24:1) and chloroform-isoamyl alcohol (24:1). DNA was precipitated with 0.1 volume of 3 M sodium acetate and 2.5 volumes of 100% isopropanol. Precipitated DNA was washed in 0.8 ml of 75% ethanol (EtOH), slightly air dried, and resuspended in 50 μl of UltraPure DNase/RNase-free distilled water (10977023; Gibco). The quality and quantity of DNA were assessed by NanoDrop (Thermo Fisher Scientific).

### RC-seq.

Genomic DNA from time points *T*_1_ to *T*_6_ for each hiPSC line was analyzed by retrotransposon capture sequencing (RC-seq), as described previously (69). Each library was constructed from 2 μg of input genomic DNA (gDNA) and sequenced in multiplex on an Illumina HiSeq 2500 instrument (Macrogen, South Korea). Fibroblast samples (time point *T*_0_) were previously analyzed by RC-seq (58). A total of 726,181,832 paired-end 2- by 150-mer reads were generated across 18 libraries (Table 1). RC-seq data were analyzed with TEBreak (https://github.com/adamewing/tebreak). Reads were aligned to the hg19 reference genome sequence using Burrows-Wheeler Aligner maximal exact match (BWA-MEM) (113) with parameters -Y and -M. Duplicate reads were marked with Picard MarkDuplicates (http://broadinstitute.github.io/picard). Candidate nonreference genome L1 insertions that were (i) detected in only one of the two hiPSC lines analyzed, (ii) absent from the matching parental fibroblasts, and (iii) did not correspond to a known nonreference germ line transposable element insertions (35, 49, 77, 79–87, 114–116) were annotated as putatively *de novo* (see Table S1 in the supplemental material). The remaining nonreference L1 insertions were annotated as polymorphic.

### PCR validation of L1 insertions.

RC-seq reads indicating putative *de novo* L1 insertions were manually inspected, and primers (Table 2) were designed to PCR amplify integration sites and identify the hallmarks of bona fide L1 retrotransposition events (117). Empty/filled-site, 5ʹ L1-genome junction, and 3ʹ L1-genome junction PCRs were performed. Primers were situated within flanking genomic DNA sequences for empty/filled-site PCRs. The same flanking primers were paired with appropriate L1-specific primers for L1-genome junction assays. Expand long-range enzyme was used for empty/filled-site PCRs using 1.75 U of Expand Long Template enzyme (04829069001; Roche), 5 μl of 5× buffer with 12.5 mM MgCl_2_, 1.25 μl of 100% dimethyl sulfoxide (DMSO), 1.25 μl 10 mM deoxynucleoside triphosphates (dNTPs), 1 μl of primer mix (25 μM each primer), 4 ng of genomic DNA template, and molecular-grade water in a final volume of 25 μl under the following PCR conditions: 92°C for 2 min, followed first by 10 cycles at 92°C for 10 s, 59°C for 15 s, and 68°C for 6.5 min and then by 30 cycles at 92°C for 2 min, 59°C for 15 s, and 68°C for 6.5 min plus 20 s of extension time per cycle, with a single extension step at 68°C for 10 min. The 5ʹ and 3ʹ L1-genome junction PCRs were performed using 2 U of MyTaq hot-start DNA polymerase (BIO-21112; Bioline), 1× PCR buffer, 1 μM each primer, 5 ng of genomic DNA template, and molecular-grade water in a final volume of 25 μl. Cycling conditions were as follows: 95°C for 2 min, followed by 35 cycles at 95°C for 30 s, 58°C for 30 s, and 72°C for 3 min, with a single extension step of 72°C for 5 min. Amplified fragments were resolved on 1% and 2% agarose gels (1× Tris-acetate-EDTA [TAE] buffer) stained with SybrSafe (Life Technologies) for empty/filled-site and 5ʹ and 3ʹ junction PCR assays, respectively, and imaged using a Typhoon FLA 9500 (GE Healthcare Life Sciences, USA). Amplicons of the expected size were excised from the gels, and DNA was extracted using a QIAquick gel extraction kit (28704; Qiagen), followed by capillary sequencing to confirm and characterize L1 insertion structural features.

### L1 genotyping and cloning.

To facilitate cloning of full-length L1 insertions, a NotI restriction enzyme sequence (5ʹ-GC/GGCC) was introduced at the 5ʹ end of each forward primer close to the L1-genome junction. Purified PCR products (500 ng) approximately 6 kbp in size were digested with NotI and Bstz17I (R3138; New England Biolabs) in 1× CutSmart buffer at 37°C for 1 h. Digestion reactions were run in 2% agarose gels (1× TAE buffer), purified by phenol-chloroform extraction, and cloned into the vector TOPO-XL PCR cloning kit (K4700-20; Life Technologies) according to the manufacturer’s instructions. Five microliters of the ligation product was used to transform One Shot TOP10 electrocompetent bacteria as per the manufacturer’s instructions. LB agar containing 0.5 μg/ml of kanamycin was used to plate bacteria, which were incubated at 37°C overnight. Single colonies were picked and transferred to 5 ml of LB liquid containing 0.5 μg/ml of kanamycin for Miniprep plasmid purification (12143; Qiagen).

To filter induced PCR mutations and distinguish possible allelic variants, at least four independent PCR products, and clones from each L1 transduction family member were capillary sequenced using 12 overlapping primer pairs (Table 2) distributed at ∼500-bp intervals covering the entire L1 sequence. Each independent clone sequence was then manually assembled and aligned with the other clones of the same element using Clustal Omega (https://www.ebi.ac.uk/Tools/msa/clustalo/). For each L1, a consensus sequence was obtained, and a mutation-free construct was reconstructed by performing multiple restriction enzyme digestions. The desired fragments were resolved in a 2% agarose gel (1× TAE buffer), purified, and ligated into a pCEP4 vector using T4 ligase in a 5:1 (insert/vector) ratio. Five microliters of the ligation product was used to transform One Shot TOP10 chemically competent bacteria (C404010; Invitrogen) as per the manufacturer’s instructions. LB agar containing 1 μg/ml of ampicillin was used to plate the bacteria, and these were incubated at 37°C overnight. Single colonies were picked and transferred to 5 ml of LB liquid containing ampicillin for Miniprep plasmid purification. To verify the fidelity of the resultant clones, these were capillary sequenced, as described above, using 12 different primers covering the entire L1 sequence.

Retrotransposition indicator plasmids termed L1.3 and L1.3 RT^−^ were generated through modification of the pCEP4 backbone of pJM101/L1.3 (14, 91) and pJM105/L1.3 (118) by removing a BgIII fragment containing the cytomegalovirus (CMV) promoter. The full L1.3 3ʹ UTR, except for a point mutation disrupting the native L1 polyadenylation signal, was reintroduced, and a PacI site was incorporated between the L1.3 3ʹ UTR and the Neo cassette (F. J. Sanchez-Luque and G. J. Faulkner, unpublished data). The mutation-free full-length transduction family members described above were then introduced into this retrotransposition indicator backbone.

DNA-binding protein motif analyses of the lineage progenitor, donor, and *de novo* L1 sequences were performed using the Catalog of Inferred Sequence Binding Preferences (CIS-BP) database (92).

### Retrotransposition assay.

HeLa-JVM cells grown in a humidified, 5% CO_2_ incubator at 37°C in high-glucose Dulbecco’s modified Eagle’s medium (DMEM) without pyruvate (11965-092; Gibco), supplemented with 10% fetal bovine serum (26400-044; Gibco), 2 mM l-glutamine, 100 U/ml penicillin, and 100 μg/ml streptomycin (10378-016; Gibco) (DMEM complete). Plasmid DNA was purified using a Midi kit (13343; Qiagen) and diluted in sterile water to 0.5 μg/μl. Cells were transfected and seeded at 5 × 10^3^ cell/well in six-well plates using FuGENE HD transfection reagent (Promega) at a ratio of 4 μl to 1 μg of plasmid DNA. Selection with G418 began 72 h after transfection and continued every 48 h for 14 days (6). Transfection efficiency assays were performed in parallel by cotransfection of pCAG-enhanced green fluorescent protein (EGFP) with L1 reporter plasmids, as described above, with 0.5 μg of each construct and 0.5 μg of pCAG-EGFP. Cells were analyzed by flow cytometry 48 h posttransfection on a Cytoflex flow cytometer (Beckman-Coulter) at the Translational Research Institute Flow Cytometry Core. The results were used to normalize the G418-resistant colony counts with the percentage of EGFP-positive cells for each L1 reporter construct obtained in the retrotransposition assay, as performed previously (118).

### L1 CpG methylation analyses.

L1-Ta subfamily-wide and L1 locus-specific bisulfite sequencing for each time point in hiPSC-CRL1502 and hiPSC-CRL2429 was performed as described previously (52). Briefly, 500 ng of gDNA was bisulfite treated using an EZ DNA Methylation Lightning kit (Zymo Research), allowing 20 min desulfonation time and eluting in a 25-μl volume. Primers L1_Bis-F and L1_Bis-R were used to amplify the L1-Ta 5ʹ UTR region containing a CpG island (Table 2), while for the L1 locus-specific reactions, L1_Bis-R was combined with one of three forward primers placed in the genomic flank of the lineage progenitor, donor, and *de novo* L1 insertions (L1_Bis-LP, L1_Bis-Donor, and L1_Bis-DN, respectively). PCRs incorporated 1 U of MyTaq hot-start DNA polymerase (BIO-21112; Bioline), 2 μl of bisulfite-treated gDNA from each sample, 1× reaction buffer, and 2 μM each primer, in a 20-μl final volume. PCR cycling conditions were as follows: 95°C for 2 min, followed by 40 cycles of 95°C for 30 s, 54°C for 30 s, and 72°C for 30 s, with a single extension step at 72°C for 5 min. Barcoded libraries were prepared from amplicons pooled by time point and sample using a TruSeq DNA PCR-free library preparation kit (FC-121-3001/2; Illumina) and subjected to multiplexed paired-end 2- by 300-mer sequencing using an Illumina MiSeq platform. Data were processed as described previously (52) and visualized using QUMA (119) with default parameters.

### Accession number(s).

RC-seq FASTQ files were deposited in the European Nucleotide Archive under accession number PRJEB27103.

## Supplementary Material

Supplemental file 1

## References

[1] Lander ES, Linton LM, Birren B, Nusbaum C, Zody MC, Baldwin J, Devon K, Dewar K, Doyle M, FitzHugh W, Funke R, Gage D, Harris K, Heaford A, Howland J, Kann L, Lehoczky J, LeVine R, McEwan P, McKernan K, Meldrim J, Mesirov JP, Miranda C, Morris W, Naylor J, Raymond C, Rosetti M, Santos R, Sheridan A, Sougnez C, Stange-Thomann Y, Stojanovic N, Subramanian A, Wyman D, Rogers J, Sulston J, Ainscough R, Beck S, Bentley D, Burton J, Clee C, Carter N, Coulson A, Deadman R, Deloukas P, Dunham A, Dunham I, Durbin R, French L, Grafham D, 2001 Initial sequencing and analysis of the human genome. Nature 409:860–921. doi:10.1038/35057062. 11237011

[2] Kazazian HH Jr, Moran JV 2017 Mobile DNA in health and disease. N Engl J Med 377:361–370. doi:10.1056/NEJMra1510092. 28745987 PMC5980640

[3] Brouha B, Schustak J, Badge RM, Lutz-Prigge S, Farley AH, Moran JV, Kazazian HH Jr. 2003 Hot L1s account for the bulk of retrotransposition in the human population. Proc Natl Acad Sci U S A 100:5280–5285. doi:10.1073/pnas.0831042100. 12682288 PMC154336

[4] Myers JS, Vincent BJ, Udall H, Watkins WS, Morrish TA, Kilroy GE, Swergold GD, Henke J, Henke L, Moran JV, Jorde LB, Batzer MA 2002 A comprehensive analysis of recently integrated human Ta L1 elements. Am J Hum Genet 71:312–326. doi:10.1086/341718. 12070800 PMC379164

[5] Mills RE, Bennett EA, Iskow RC, Devine SE 2007 Which transposable elements are active in the human genome? Trends Genet 23:183–191. doi:10.1016/j.tig.2007.02.006. 17331616

[6] Wei W, Gilbert N, Ooi SL, Lawler JF, Ostertag EM, Kazazian HH, Boeke JD, Moran JV 2001 Human L1 retrotransposition: cis preference versus trans complementation. Mol Cell Biol 21:1429–1439. doi:10.1128/MCB.21.4.1429-1439.2001. 11158327 PMC99594

[7] Boeke JD, Garfinkel DJ, Styles CA, Fink GR 1985 Ty elements transpose through an RNA intermediate. Cell 40:491–500. doi:10.1016/0092-8674(85)90197-7. 2982495

[8] Moran JV, Holmes SE, Naas TP, DeBerardinis RJ, Boeke JD, Kazazian HH J.r 1996 High frequency retrotransposition in cultured mammalian cells. Cell 87:917–927. doi:10.1016/S0092-8674(00)81998-4. 8945518

[9] Luan DD, Korman MH, Jakubczak JL, Eickbush TH 1993 Reverse transcription of R2Bm RNA is primed by a nick at the chromosomal target site: a mechanism for non-LTR retrotransposition. Cell 72:595–605. doi:10.1016/0092-8674(93)90078-5. 7679954

[10] Denli AM, Narvaiza I, Kerman BE, Pena M, Benner C, Marchetto MC, Diedrich JK, Aslanian A, Ma J, Moresco JJ, Moore L, Hunter T, Saghatelian A, Gage FH 2015 Primate-specific ORF0 contributes to retrotransposon-mediated diversity. Cell 163:583–593. doi:10.1016/j.cell.2015.09.025. 26496605

[11] Speek M 2001 Antisense promoter of human L1 retrotransposon drives transcription of adjacent cellular genes. Mol Cell Biol 21:1973–1985. doi:10.1128/MCB.21.6.1973-1985.2001. 11238933 PMC86790

[12] Scott AF, Schmeckpeper BJ, Abdelrazik M, Comey CT, O'Hara B, Rossiter JP, Cooley T, Heath P, Smith KD, Margolet L 1987 Origin of the human L1 elements: proposed progenitor genes deduced from a consensus DNA sequence. Genomics 1:113–125. doi:10.1016/0888-7543(87)90003-6. 3692483 PMC7135745

[13] Doucet AJ, Wilusz JE, Miyoshi T, Liu Y, Moran JV 2015 A 3' poly(A) tract is required for LINE-1 retrotransposition. Mol Cell 60:728–741. doi:10.1016/j.molcel.2015.10.012. 26585388 PMC4671821

[14] Dombroski BA, Mathias SL, Nanthakumar E, Scott AF, Kazazian HH. Jr, 1991 Isolation of an active human transposable element. Science 254:1805–1808. doi:10.1126/science.1662412. 1662412

[15] Mathias SL, Scott AF, Kazazian HH Jr, Boeke JD, Gabriel A 1991 Reverse transcriptase encoded by a human transposable element. Science 254:1808–1810. doi:10.1126/science.1722352. 1722352

[16] Feng Q, Moran JV, Kazazian HH Jr, Boeke JD 1996 Human L1 retrotransposon encodes a conserved endonuclease required for retrotransposition. Cell 87:905–916. doi:10.1016/S0092-8674(00)81997-2. 8945517

[17] Jurka J 1997 Sequence patterns indicate an enzymatic involvement in integration of mammalian retroposons. Proc Natl Acad Sci U S A 94:1872–1877. doi:10.1073/pnas.94.5.1872. 9050872 PMC20010

[18] Gilbert N, Lutz S, Morrish TA, Moran JV 2005 Multiple fates of L1 retrotransposition intermediates in cultured human cells. Mol Cell Biol 25:7780–7795. doi:10.1128/MCB.25.17.7780-7795.2005. 16107723 PMC1190285

[19] Grimaldi G, Skowronski J, Singer MF 1984 Defining the beginning and end of KpnI family segments. EMBO J 3:1753–1759. doi:10.1002/j.1460-2075.1984.tb02042.x. 6090124 PMC557592

[20] Ahl V, Keller H, Schmidt S, Weichenrieder O 2015 Retrotransposition and crystal structure of an Alu RNP in the ribosome-stalling conformation. Mol Cell 60:715–727. doi:10.1016/j.molcel.2015.10.003. 26585389

[21] Esnault C, Maestre J, Heidmann T 2000 Human LINE retrotransposons generate processed pseudogenes. Nat Genet 24:363–367. doi:10.1038/74184. 10742098

[22] Dewannieux M, Esnault C, Heidmann T 2003 LINE-mediated retrotransposition of marked Alu sequences. Nat Genet 35:41–48. doi:10.1038/ng1223. 12897783

[23] Raiz J, Damert A, Chira S, Held U, Klawitter S, Hamdorf M, Lower J, Stratling WH, Lower R, Schumann GG 2012 The non-autonomous retrotransposon SVA is trans-mobilized by the human LINE-1 protein machinery. Nucleic Acids Res 40:1666–1683. doi:10.1093/nar/gkr863. 22053090 PMC3287187

[24] Hancks DC, Goodier JL, Mandal PK, Cheung LE, Kazazian HH Jr. 2011 Retrotransposition of marked SVA elements by human L1s in cultured cells. Hum Mol Genet 20:3386–3400. doi:10.1093/hmg/ddr245. 21636526 PMC3153304

[25] Swergold GD 1990 Identification, characterization, and cell specificity of a human LINE-1 promoter. Mol Cell Biol 10:6718–6729. doi:10.1128/MCB.10.12.6718. 1701022 PMC362950

[26] Hata K, Sakaki Y 1997 Identification of critical CpG sites for repression of L1 transcription by DNA methylation. Gene 189:227–234. doi:10.1016/S0378-1119(96)00856-6. 9168132

[27] Castro-Diaz N, Ecco G, Coluccio A, Kapopoulou A, Yazdanpanah B, Friedli M, Duc J, Jang SM, Turelli P, Trono D 2014 Evolutionally dynamic L1 regulation in embryonic stem cells. Genes Dev 28:1397–1409. doi:10.1101/gad.241661.114. 24939876 PMC4083085

[28] de la Rica L, Deniz O, Cheng KC, Todd CD, Cruz C, Houseley J, Branco MR 2016 TET-dependent regulation of retrotransposable elements in mouse embryonic stem cells. Genome Biol 17:234. doi:10.1186/s13059-016-1096-8. 27863519 PMC5116139

[29] Walter M, Teissandier A, Perez-Palacios R, Bourc'his D 2016 An epigenetic switch ensures transposon repression upon dynamic loss of DNA methylation in embryonic stem cells. Elife 5:e11418. doi:10.7554/eLife.11418. 26814573 PMC4769179

[30] Robbez-Masson L, Tie CHC, Conde L, Tunbak H, Husovsky C, Tchasovnikarova IA, Timms RT, Herrero J, Lehner PJ, Rowe HM 2018 The HUSH complex cooperates with TRIM28 to repress young retrotransposons and new genes. Genome Res 28:836–845. doi:10.1101/gr.228171.117. 29728366 PMC5991525

[31] Thayer RE, Singer MF, Fanning TG 1993 Undermethylation of specific LINE-1 sequences in human cells producing a LINE-1-encoded protein. Gene 133:273–277. doi:10.1016/0378-1119(93)90651-I. 7693554

[32] Beck CR, Garcia-Perez JL, Badge RM, Moran JV 2011 LINE-1 elements in structural variation and disease. Annu Rev Genomics Hum Genet 12:187–215. doi:10.1146/annurev-genom-082509-141802. 21801021 PMC4124830

[33] Kuwabara T, Hsieh J, Muotri A, Yeo G, Warashina M, Lie DC, Moore L, Nakashima K, Asashima M, Gage FH 2009 Wnt-mediated activation of NeuroD1 and retro-elements during adult neurogenesis. Nat Neurosci 12:1097–1105. doi:10.1038/nn.2360. 19701198 PMC2764260

[34] Goodier JL 2016 Restricting retrotransposons: a review. Mob DNA 7:16. doi:10.1186/s13100-016-0070-z. 27525044 PMC4982230

[35] Beck CR, Collier P, Macfarlane C, Malig M, Kidd JM, Eichler EE, Badge RM, Moran JV 2010 LINE-1 retrotransposition activity in human genomes. Cell 141:1159–1170. doi:10.1016/j.cell.2010.05.021. 20602998 PMC3013285

[36] Ostertag EM, Prak ET, DeBerardinis RJ, Moran JV, Kazazian HH Jr. 2000 Determination of L1 retrotransposition kinetics in cultured cells. Nucleic Acids Res 28:1418–1423. doi:10.1093/nar/28.6.1418. 10684937 PMC111040

[37] Gardner EJ, Lam VK, Harris DN, Chuang NT, Scott EC, Pittard WS, Mills RE, Genomes Project C, Devine SE 2017 The Mobile Element Locator Tool (MELT): population-scale mobile element discovery and biology. Genome Res 27:1916–1929. doi:10.1101/gr.218032.116. 28855259 PMC5668948

[38] Seleme MC, Vetter MR, Cordaux R, Bastone L, Batzer MA, Kazazian HH Jr. 2006 Extensive individual variation in L1 retrotransposition capability contributes to human genetic diversity. Proc Natl Acad Sci U S A 103:6611–6616. doi:10.1073/pnas.0601324103. 16618923 PMC1458931

[39] Lutz SM, Vincent BJ, Kazazian HH Jr, Batzer MA, Moran JV 2003 Allelic heterogeneity in LINE-1 retrotransposition activity. Am J Hum Genet 73:1431–1437. doi:10.1086/379744. 14610717 PMC1180405

[40] Scott EC, Gardner EJ, Masood A, Chuang NT, Vertino PM, Devine SE 2016 A hot L1 retrotransposon evades somatic repression and initiates human colorectal cancer. Genome Res 26:745–755. doi:10.1101/gr.201814.115. 27197217 PMC4889970

[41] Athanikar JN, Badge RM, Moran JV 2004 A YY1-binding site is required for accurate human LINE-1 transcription initiation. Nucleic Acids Res 32:3846–3855. doi:10.1093/nar/gkh698. 15272086 PMC506791

[42] Symer DE, Connelly C, Szak ST, Caputo EM, Cost GJ, Parmigiani G, Boeke JD 2002 Human l1 retrotransposition is associated with genetic instability in vivo. Cell 110:327–338. doi:10.1016/S0092-8674(02)00839-5. 12176320

[43] Larson PA, Moldovan JB, Jasti N, Kidd JM, Beck CR, Moran JV 2018 Spliced integrated retrotransposed element (SpIRE) formation in the human genome. PLoS Biol 16:e2003067. doi:10.1371/journal.pbio.2003067. 29505568 PMC5860796

[44] Macfarlane CM, Collier P, Rahbari R, Beck CR, Wagstaff JF, Igoe S, Moran JV, Badge RM 2013 Transduction-specific ATLAS reveals a cohort of highly active L1 retrotransposons in human populations. Hum Mutat 34:974–985. doi:10.1002/humu.22327. 23553801 PMC3880804

[45] Goodier JL, Ostertag EM, Kazazian HH Jr. 2000 Transduction of 3'-flanking sequences is common in L1 retrotransposition. Hum Mol Genet 9:653–657. doi:10.1093/hmg/9.4.653. 10699189

[46] Moran JV 1999 Human L1 retrotransposition: insights and peculiarities learned from a cultured cell retrotransposition assay. Genetica 107:39–51. doi:10.1023/A:1004035023356. 10952196

[47] Moran JV, DeBerardinis RJ, Kazazian HH Jr. 1999 Exon shuffling by L1 retrotransposition. Science 283:1530–1534. doi:10.1126/science.283.5407.1530. 10066175

[48] Pickeral OK, Makałowski W, Boguski MS, Boeke JD 2000 Frequent human genomic DNA transduction driven by LINE-1 retrotransposition. Genome Res 10:411–415. doi:10.1101/gr.10.4.411. 10779482 PMC310862

[49] Tubio JMC, Li Y, Ju YS, Martincorena I, Cooke SL, Tojo M, Gundem G, Pipinikas CP, Zamora J, Raine K, Menzies A, Roman-Garcia P, Fullam A, Gerstung M, Shlien A, Tarpey PS, Papaemmanuil E, Knappskog S, Van Loo P, Ramakrishna M, Davies HR, Marshall J, Wedge DC, Teague JW, Butler AP, Nik-Zainal S, Alexandrov L, Behjati S, Yates LR, Bolli N, Mudie L, Hardy C, Martin S, McLaren S, O'Meara S, Anderson E, Maddison M, Gamble S, Foster C, Warren AY, Whitaker H, Brewer D, Eeles R, Cooper C, Neal D, Lynch AG, Visakorpi T, Isaacs WB, Veer LV, Caldas C, 2014 Mobile DNA in cancer. Extensive transduction of nonrepetitive DNA mediated by L1 retrotransposition in cancer genomes. Science 345:1251343. doi:10.1126/science.1251343. 25082706 PMC4380235

[50] Solyom S, Ewing AD, Hancks DC, Takeshima Y, Awano H, Matsuo M, Kazazian HH Jr. 2012 Pathogenic orphan transduction created by a nonreference LINE-1 retrotransposon. Hum Mutat 33:369–371. doi:10.1002/humu.21663. 22095564 PMC3258325

[51] Brouha B, Meischl C, Ostertag E, de Boer M, Zhang Y, Neijens H, Roos D, Kazazian HH Jr. 2002 Evidence consistent with human L1 retrotransposition in maternal meiosis I. Am J Hum Genet 71:327–336. doi:10.1086/341722. 12094329 PMC379165

[52] Nguyen THM, Carreira PE, Sanchez-Luque FJ, Schauer SN, Fagg AC, Richardson SR, Davies CM, Jesuadian JS, Kempen MHC, Troskie RL, James C, Beaven EA, Wallis TP, Coward JIG, Chetty NP, Crandon AJ, Venter DJ, Armes JE, Perrin LC, Hooper JD, Ewing AD, Upton KR, Faulkner GJ 2018 L1 retrotransposon heterogeneity in ovarian tumor cell evolution. Cell Rep 23:3730–3740. doi:10.1016/j.celrep.2018.05.090. 29949758

[53] Holmes SE, Dombroski BA, Krebs CM, Boehm CD, Kazazian HH Jr. 1994 A new retrotransposable human L1 element from the LRE2 locus on chromosome 1q produces a chimaeric insertion. Nat Genet 7:143–148. doi:10.1038/ng0694-143. 7920631

[54] Szak ST, Pickeral OK, Landsman D, Boeke JD 2003 Identifying related L1 retrotransposons by analyzing 3' transduced sequences. Genome Biol 4:R30. doi:10.1186/gb-2003-4-5-r30. 12734010 PMC156586

[55] Richardson SR, Gerdes P, Gerhardt DJ, Sanchez-Luque FJ, Bodea GO, Munoz-Lopez M, Jesuadian JS, Kempen MHC, Carreira PE, Jeddeloh JA, Garcia-Perez JL, Kazazian HH Jr, Ewing AD, Faulkner GJ 2017 Heritable L1 retrotransposition in the mouse primordial germline and early embryo. Genome Res 27:1395–1405. doi:10.1101/gr.219022.116. 28483779 PMC5538555

[56] Richardson SR, Faulkner GJ 2018 Heritable L1 retrotransposition events during development: understanding their origins. Bioessays 40:e1700189. doi:10.1002/bies.201700189. 29709066 PMC6681178

[57] van den Hurk JA, Meij IC, Seleme MC, Kano H, Nikopoulos K, Hoefsloot LH, Sistermans EA, de Wijs IJ, Mukhopadhyay A, Plomp AS, de Jong PT, Kazazian HH, Cremers FP 2007 L1 retrotransposition can occur early in human embryonic development. Hum Mol Genet 16:1587–1592. doi:10.1093/hmg/ddm108. 17483097

[58] Klawitter S, Fuchs NV, Upton KR, Muñoz-Lopez M, Shukla R, Wang J, Garcia-Cañadas M, Lopez-Ruiz C, Gerhardt DJ, Sebe A, Grabundzija I, Merkert S, Gerdes P, Pulgarin JA, Bock A, Held U, Witthuhn A, Haase A, Sarkadi B, Löwer J, Wolvetang EJ, Martin U, Ivics Z, Izsvák Z, Garcia-Perez JL, Faulkner GJ, Schumann GG 2016 Reprogramming triggers endogenous L1 and Alu retrotransposition in human induced pluripotent stem cells. Nat Commun 7:10286. doi:10.1038/ncomms10286. 26743714 PMC4729875

[59] Garcia-Perez JL, Marchetto MC, Muotri AR, Coufal NG, Gage FH, O'Shea KS, Moran JV 2007 LINE-1 retrotransposition in human embryonic stem cells. Hum Mol Genet 16:1569–1577. doi:10.1093/hmg/ddm105. 17468180

[60] Wissing S, Montano M, Garcia-Perez JL, Moran JV, Greene WC 2011 Endogenous APOBEC3B restricts LINE-1 retrotransposition in transformed cells and human embryonic stem cells. J Biol Chem 286:36427–36437. doi:10.1074/jbc.M111.251058. 21878639 PMC3196128

[61] Wissing S, Munoz-Lopez M, Macia A, Yang Z, Montano M, Collins W, Garcia-Perez JL, Moran JV, Greene WC 2012 Reprogramming somatic cells into iPS cells activates LINE-1 retroelement mobility. Hum Mol Genet 21:208–218. doi:10.1093/hmg/ddr455. 21989055 PMC3235014

[62] Macia A, Munoz-Lopez M, Cortes JL, Hastings RK, Morell S, Lucena-Aguilar G, Marchal JA, Badge RM, Garcia-Perez JL 2011 Epigenetic control of retrotransposon expression in human embryonic stem cells. Mol Cell Biol 31:300–316. doi:10.1128/MCB.00561-10. 21041477 PMC3019972

[63] Macia A, Widmann TJ, Heras SR, Ayllon V, Sanchez L, Benkaddour-Boumzaouad M, Munoz-Lopez M, Rubio A, Amador-Cubero S, Blanco-Jimenez E, Garcia-Castro J, Menendez P, Ng P, Muotri AR, Goodier JL, Garcia-Perez JL 2017 Engineered LINE-1 retrotransposition in nondividing human neurons. Genome Res 27:335–348. doi:10.1101/gr.206805.116. 27965292 PMC5340962

[64] Kano H, Godoy I, Courtney C, Vetter MR, Gerton GL, Ostertag EM, Kazazian HH Jr. 2009 L1 retrotransposition occurs mainly in embryogenesis and creates somatic mosaicism. Genes Dev 23:1303–1312. doi:10.1101/gad.1803909. 19487571 PMC2701581

[65] Faulkner GJ, Garcia-Perez JL 2017 L1 mosaicism in mammals: extent, effects, and evolution. Trends Genet 33:802–816. doi:10.1016/j.tig.2017.07.004. 28797643

[66] An W, Han JS, Wheelan SJ, Davis ES, Coombes CE, Ye P, Triplett C, Boeke JD 2006 Active retrotransposition by a synthetic L1 element in mice. Proc Natl Acad Sci U S A 103:18662–18667. doi:10.1073/pnas.0605300103. 17124176 PMC1693719

[67] Coufal NG, Garcia-Perez JL, Peng GE, Yeo GW, Mu Y, Lovci MT, Morell M, O’Shea KS, Moran JV, Gage FH 2009 L1 retrotransposition in human neural progenitor cells. Nature 460:1127–1131. doi:10.1038/nature08248. 19657334 PMC2909034

[68] Faulkner GJ, Billon V 2018 L1 retrotransposition in the soma: a field jumping ahead. Mob DNA 9:22. doi:10.1186/s13100-018-0128-1. 30002735 PMC6035798

[69] Upton KR, Gerhardt DJ, Jesuadian JS, Richardson SR, Sanchez-Luque FJ, Bodea GO, Ewing AD, Salvador-Palomeque C, van der Knaap MS, Brennan PM, Vanderver A, Faulkner GJ 2015 Ubiquitous L1 mosaicism in hippocampal neurons. Cell 161:228–239. doi:10.1016/j.cell.2015.03.026. 25860606 PMC4398972

[70] Baillie JK, Barnett MW, Upton KR, Gerhardt DJ, Richmond TA, De Sapio F, Brennan PM, Rizzu P, Smith S, Fell M, Talbot RT, Gustincich S, Freeman TC, Mattick JS, Hume DA, Heutink P, Carninci P, Jeddeloh JA, Faulkner GJ 2011 Somatic retrotransposition alters the genetic landscape of the human brain. Nature 479:534–537. doi:10.1038/nature10531. 22037309 PMC3224101

[71] Evrony GD, Cai X, Lee E, Hills LB, Elhosary PC, Lehmann HS, Parker JJ, Atabay KD, Gilmore EC, Poduri A, Park PJ, Walsh CA 2012 Single-neuron sequencing analysis of L1 retrotransposition and somatic mutation in the human brain. Cell 151:483–496. doi:10.1016/j.cell.2012.09.035. 23101622 PMC3567441

[72] Evrony GD, Lee E, Mehta BK, Benjamini Y, Johnson RM, Cai X, Yang L, Haseley P, Lehmann HS, Park PJ, Walsh CA 2015 Cell lineage analysis in human brain using endogenous retroelements. Neuron 85:49–59. doi:10.1016/j.neuron.2014.12.028. 25569347 PMC4299461

[73] Hazen JL, Faust GG, Rodriguez AR, Ferguson WC, Shumilina S, Clark RA, Boland MJ, Martin G, Chubukov P, Tsunemoto RK, Torkamani A, Kupriyanov S, Hall IM, Baldwin KK 2016 The complete genome sequences, unique mutational spectra, and developmental potency of adult neurons revealed by cloning. Neuron 89:1223–1236. doi:10.1016/j.neuron.2016.02.004. 26948891 PMC4795965

[74] Erwin JA, Paquola AC, Singer T, Gallina I, Novotny M, Quayle C, Bedrosian TA, Alves FI, Butcher CR, Herdy JR, Sarkar A, Lasken RS, Muotri AR, Gage FH 2016 L1-associated genomic regions are deleted in somatic cells of the healthy human brain. Nat Neurosci 19:1583–1591. doi:10.1038/nn.4388. 27618310 PMC5127747

[75] Muotri AR, Chu VT, Marchetto MC, Deng W, Moran JV, Gage FH 2005 Somatic mosaicism in neuronal precursor cells mediated by L1 retrotransposition. Nature 435:903–910. doi:10.1038/nature03663. 15959507

[76] Briggs JA, Sun J, Shepherd J, Ovchinnikov DA, Chung TL, Nayler SP, Kao LP, Morrow CA, Thakar NY, Soo SY, Peura T, Grimmond S, Wolvetang EJ 2013 Integration-free induced pluripotent stem cells model genetic and neural developmental features of down syndrome etiology. Stem Cells 31:467–478. doi:10.1002/stem.1297. 23225669

[77] Shukla R, Upton KR, Munoz-Lopez M, Gerhardt DJ, Fisher ME, Nguyen T, Brennan PM, Baillie JK, Collino A, Ghisletti S, Sinha S, Iannelli F, Radaelli E, Dos Santos A, Rapoud D, Guettier C, Samuel D, Natoli G, Carninci P, Ciccarelli FD, Garcia-Perez JL, Faivre J, Faulkner GJ 2013 Endogenous retrotransposition activates oncogenic pathways in hepatocellular carcinoma. Cell 153:101–111. doi:10.1016/j.cell.2013.02.032. 23540693 PMC3898742

[78] Schauer SN, Carreira PE, Shukla R, Gerhardt DJ, Gerdes P, Sanchez-Luque FJ, Nicoli P, Kindlova M, Ghisletti S, Santos AD, Rapoud D, Samuel D, Faivre J, Ewing AD, Richardson SR, Faulkner GJ 2018 L1 retrotransposition is a common feature of mammalian hepatocarcinogenesis. Genome Res 28:639–653. doi:10.1101/gr.226993.117. 29643204 PMC5932605

[79] Ewing AD, Kazazian HH Jr. 2011 Whole-genome resequencing allows detection of many rare LINE-1 insertion alleles in humans. Genome Res 21:985–990. doi:10.1101/gr.114777.110. 20980553 PMC3106331

[80] Ewing AD, Kazazian HH Jr. 2010 High-throughput sequencing reveals extensive variation in human-specific L1 content in individual human genomes. Genome Res 20:1262–1270. doi:10.1101/gr.106419.110. 20488934 PMC2928504

[81] Lee E, Iskow R, Yang L, Gokcumen O, Haseley P, Luquette LJ, Lohr JG, Harris CC, Ding L, Wilson RK, Wheeler DA, Gibbs RA, Kucherlapati R, Lee C, Kharchenko PV, Park PJ, The Cancer Genome Atlas Research Network 2012 Landscape of somatic retrotransposition in human cancers. Science 337:967–971. doi:10.1126/science.1222077. 22745252 PMC3656569

[82] Iskow RC, McCabe MT, Mills RE, Torene S, Pittard WS, Neuwald AF, Van Meir EG, Vertino PM, Devine SE 2010 Natural mutagenesis of human genomes by endogenous retrotransposons. Cell 141:1253–1261. doi:10.1016/j.cell.2010.05.020. 20603005 PMC2943760

[83] Helman E, Lawrence MS, Stewart C, Sougnez C, Getz G, Meyerson M 2014 Somatic retrotransposition in human cancer revealed by whole-genome and exome sequencing. Genome Res 24:1053–1063. doi:10.1101/gr.163659.113. 24823667 PMC4079962

[84] Kuhn A, Ong YM, Cheng CY, Wong TY, Quake SR, Burkholder WF 2014 Linkage disequilibrium and signatures of positive selection around LINE-1 retrotransposons in the human genome. Proc Natl Acad Sci U S A 111:8131–8136. doi:10.1073/pnas.1401532111. 24847061 PMC4050588

[85] Stewart C, Kural D, Stromberg MP, Walker JA, Konkel MK, Stutz AM, Urban AE, Grubert F, Lam HY, Lee WP, Busby M, Indap AR, Garrison E, Huff C, Xing J, Snyder MP, Jorde LB, Batzer MA, Korbel JO, Marth GT, 1000 Genomes Project 2011 A comprehensive map of mobile element insertion polymorphisms in humans. PLoS Genet 7:e1002236. doi:10.1371/journal.pgen.1002236. 21876680 PMC3158055

[86] Sudmant PH, Rausch T, Gardner EJ, Handsaker RE, Abyzov A, Huddleston J, Zhang Y, Ye K, Jun G, Fritz MH-Y, Konkel MK, Malhotra A, Stütz AM, Shi X, Casale FP, Chen J, Hormozdiari F, Dayama G, Chen K, Malig M, Chaisson MJP, Walter K, Meiers S, Kashin S, Garrison E, Auton A, Lam HYK, Mu XJ, Alkan C, Antaki D, Bae T, Cerveira E, Chines P, Chong Z, Clarke L, Dal E, Ding L, Emery S, Fan X, Gujral M, Kahveci F, Kidd JM, Kong Y, Lameijer E-W, McCarthy S, Flicek P, Gibbs RA, Marth G, Mason CE, Menelaou A, 2015 An integrated map of structural variation in 2,504 human genomes. Nature 526:75–81. doi:10.1038/nature15394. 26432246 PMC4617611

[87] Wang J, Song L, Grover D, Azrak S, Batzer MA, Liang P 2006 dbRIP: a highly integrated database of retrotransposon insertion polymorphisms in humans. Hum Mutat 27:323–329. doi:10.1002/humu.20307. 16511833 PMC1855216

[88] Dombroski BA, Scott AF, Kazazian HH Jr, 1993 Two additional potential retrotransposons isolated from a human L1 subfamily that contains an active retrotransposable element. Proc Natl Acad Sci U S A 90:6513–6517. doi:10.1073/pnas.90.14.6513. 8393568 PMC46962

[89] Lavie L, Maldener E, Brouha B, Meese EU, Mayer J 2004 The human L1 promoter: variable transcription initiation sites and a major impact of upstream flanking sequence on promoter activity. Genome Res 14:2253–2260. doi:10.1101/gr.2745804. 15520289 PMC525683

[90] Kopera HC, Larson PA, Moldovan JB, Richardson SR, Liu Y, Moran JV 2016 LINE-1 cultured cell retrotransposition assay. Methods Mol Biol 1400:139–156. doi:10.1007/978-1-4939-3372-3_10. 26895052 PMC5070806

[91] Sassaman DM, Dombroski BA, Moran JV, Kimberland ML, Naas TP, DeBerardinis RJ, Gabriel A, Swergold GD, Kazazian HH Jr. 1997 Many human L1 elements are capable of retrotransposition. Nat Genet 16:37–43. doi:10.1038/ng0597-37. 9140393

[92] Weirauch MT, Yang A, Albu M, Cote AG, Montenegro-Montero A, Drewe P, Najafabadi HS, Lambert SA, Mann I, Cook K, Zheng H, Goity A, van Bakel H, Lozano JC, Galli M, Lewsey MG, Huang E, Mukherjee T, Chen X, Reece-Hoyes JS, Govindarajan S, Shaulsky G, Walhout AJM, Bouget FY, Ratsch G, Larrondo LF, Ecker JR, Hughes TR 2014 Determination and inference of eukaryotic transcription factor sequence specificity. Cell 158:1431–1443. doi:10.1016/j.cell.2014.08.009. 25215497 PMC4163041

[93] Pierrou S, Hellqvist M, Samuelsson L, Enerback S, Carlsson P 1994 Cloning and characterization of seven human forkhead proteins: binding site specificity and DNA bending. EMBO J 13:5002–5012. doi:10.1002/j.1460-2075.1994.tb06827.x. 7957066 PMC395442

[94] Zaret KS, Carroll JS 2011 Pioneer transcription factors: establishing competence for gene expression. Genes Dev 25:2227–2241. doi:10.1101/gad.176826.111. 22056668 PMC3219227

[95] Allenby G, Bocquel MT, Saunders M, Kazmer S, Speck J, Rosenberger M, Lovey A, Kastner P, Grippo JF, Chambon P 1993 Retinoic acid receptors and retinoid X receptors: interactions with endogenous retinoic acids. Proc Natl Acad Sci U S A 90:30–34. doi:10.1073/pnas.90.1.30. 8380496 PMC45593

[96] Muotri AR, Marchetto MC, Coufal NG, Oefner R, Yeo G, Nakashima K, Gage FH 2010 L1 retrotransposition in neurons is modulated by MeCP2. Nature 468:443–446. doi:10.1038/nature09544. 21085180 PMC3059197

[97] Suzuki M, Yamada T, Kihara-Negishi F, Sakurai T, Hara E, Tenen DG, Hozumi N, Oikawa T 2006 Site-specific DNA methylation by a complex of PU.1 and Dnmt3a/b. Oncogene 25:2477–2488. doi:10.1038/sj.onc.1209272. 16331260

[98] Yu F, Zingler N, Schumann G, Stratling WH 2001 Methyl-CpG-binding protein 2 represses LINE-1 expression and retrotransposition but not Alu transcription. Nucleic Acids Res 29:4493–4501. doi:10.1093/nar/29.21.4493. 11691937 PMC60185

[99] Durruthy-Durruthy J, Sebastiano V, Wossidlo M, Cepeda D, Cui J, Grow EJ, Davila J, Mall M, Wong WH, Wysocka J, Au KF, Reijo Pera RA 2016 The primate-specific noncoding RNA HPAT5 regulates pluripotency during human preimplantation development and nuclear reprogramming. Nat Genet 48:44–52. doi:10.1038/ng.3449. 26595768 PMC4827613

[100] Faulkner GJ, Kimura Y, Daub CO, Wani S, Plessy C, Irvine KM, Schroder K, Cloonan N, Steptoe AL, Lassmann T, Waki K, Hornig N, Arakawa T, Takahashi H, Kawai J, Forrest AR, Suzuki H, Hayashizaki Y, Hume DA, Orlando V, Grimmond SM, Carninci P 2009 The regulated retrotransposon transcriptome of mammalian cells. Nat Genet 41:563–571. doi:10.1038/ng.368. 19377475

[101] Fort A, Hashimoto K, Yamada D, Salimullah M, Keya CA, Saxena A, Bonetti A, Voineagu I, Bertin N, Kratz A, Noro Y, Wong C-H, de Hoon M, Andersson R, Sandelin A, Suzuki H, Wei C-L, Koseki H, Hasegawa Y, Forrest ARR, Carninci P 2014 Deep transcriptome profiling of mammalian stem cells supports a regulatory role for retrotransposons in pluripotency maintenance. Nat Genet 46:558–566. doi:10.1038/ng.2965. 24777452

[102] Gerdes P, Richardson SR, Mager DL, Faulkner GJ 2016 Transposable elements in the mammalian embryo: pioneers surviving through stealth and service. Genome Biol 17:100. doi:10.1186/s13059-016-0965-5. 27161170 PMC4862087

[103] Lu X, Sachs F, Ramsay L, Jacques PE, Goke J, Bourque G, Ng HH 2014 The retrovirus HERVH is a long noncoding RNA required for human embryonic stem cell identity. Nat Struct Mol Biol 21:423–425. doi:10.1038/nsmb.2799. 24681886

[104] Tchenio T, Casella JF, Heidmann T 2000 Members of the SRY family regulate the human LINE retrotransposons. Nucleic Acids Res 28:411–415. doi:10.1093/nar/28.2.411. 10606637 PMC102531

[105] Yang N, Zhang L, Zhang Y, Kazazian HH Jr. 2003 An important role for RUNX3 in human L1 transcription and retrotransposition. Nucleic Acids Res 31:4929–4940. doi:10.1093/nar/gkg663. 12907736 PMC169909

[106] Tapia N, Scholer HR 2016 Molecular obstacles to clinical translation of iPSCs. Cell Stem Cell 19:298–309. doi:10.1016/j.stem.2016.06.017. 27452174

[107] Rowitch DH, Kriegstein AR 2010 Developmental genetics of vertebrate glial-cell specification. Nature 468:214–222. doi:10.1038/nature09611. 21068830

[108] Miller FD, Gauthier AS 2007 Timing is everything: making neurons versus glia in the developing cortex. Neuron 54:357–369. doi:10.1016/j.neuron.2007.04.019. 17481390

[109] Sun YE, Martinowich K, Ge W 2003 Making and repairing the mammalian brain—signaling toward neurogenesis and gliogenesis. Semin Cell Dev Biol 14:161–168. doi:10.1016/S1084-9521(03)00007-7. 12948350

[110] Lister R, Mukamel EA, Nery JR, Urich M, Puddifoot CA, Johnson ND, Lucero J, Huang Y, Dwork AJ, Schultz MD, Yu M, Tonti-Filippini J, Heyn H, Hu S, Wu JC, Rao A, Esteller M, He C, Haghighi FG, Sejnowski TJ, Behrens MM, Ecker JR 2013 Global epigenomic reconfiguration during mammalian brain development. Science 341:1237905. doi:10.1126/science.1237905. 23828890 PMC3785061

[111] Papp B, Plath K 2013 Epigenetics of reprogramming to induced pluripotency. Cell 152:1324–1343. doi:10.1016/j.cell.2013.02.043. 23498940 PMC3602907

[112] Shi Y, Kirwan P, Livesey FJ 2012 Directed differentiation of human pluripotent stem cells to cerebral cortex neurons and neural networks. Nat Protoc 7:1836–1846. doi:10.1038/nprot.2012.116. 22976355

[113] Li H 2013 Aligning sequence reads, clone sequences and assembly contigs with BWA-MEM. arXiv 1303.3997 [q-bio.GN] https://arxiv.org/abs/1303.3997.

[114] Hormozdiari F, Alkan C, Ventura M, Hajirasouliha I, Malig M, Hach F, Yorukoglu D, Dao P, Bakhshi M, Sahinalp SC, Eichler EE 2011 Alu repeat discovery and characterization within human genomes. Genome Res 21:840–849. doi:10.1101/gr.115956.110. 21131385 PMC3106317

[115] Witherspoon DJ, Xing J, Zhang Y, Watkins WS, Batzer MA, Jorde LB 2010 Mobile element scanning (ME-Scan) by targeted high-throughput sequencing. BMC Genomics 11:410. doi:10.1186/1471-2164-11-410. 20591181 PMC2996938

[116] Witherspoon DJ, Zhang Y, Xing J, Watkins WS, Ha H, Batzer MA, Jorde LB 2013 Mobile element scanning (ME-Scan) identifies thousands of novel Alu insertions in diverse human populations. Genome Res 23:1170–1181. doi:10.1101/gr.148973.112. 23599355 PMC3698510

[117] Richardson SR, Morell S, Faulkner GJ 2014 L1 retrotransposons and somatic mosaicism in the brain. Annu Rev Genet 48:1–27. doi:10.1146/annurev-genet-120213-092412. 25036377

[118] Wei W, Morrish TA, Alisch RS, Moran JV 2000 A transient assay reveals that cultured human cells can accommodate multiple LINE-1 retrotransposition events. Anal Biochem 284:435–438. doi:10.1006/abio.2000.4675. 10964437

[119] Kumaki Y, Oda M, Okano M 2008 QUMA: quantification tool for methylation analysis. Nucleic Acids Res 36:W170–W175. doi:10.1093/nar/gkn294. 18487274 PMC2447804

